# Environmental variables and genome-environment interactions predicting IBD diagnosis in large UK cohort

**DOI:** 10.1038/s41598-022-13222-0

**Published:** 2022-06-28

**Authors:** Alan Z. Yang, Luke Jostins-Dean

**Affiliations:** 1 New College, Oxford, UK; 2 Kennedy Institute of Rheumatology, University of Oxford, Roosevelt Drive, Headington, OX3 7FY Oxfordshire UK

**Keywords:** Risk factors, Epidemiology, Inflammatory bowel disease

## Abstract

A combination of genetic susceptibility and environmental exposure is thought to cause inflammatory bowel disease (IBD), but the non-genetic component remains poorly characterized. We therefore undertook a search for environmental variables and gene-environment interactions associated with future IBD diagnosis in a large UK cohort. Using self-report and electronic health records, we identified 1946 Crohn’s disease (CD) and 3715 ulcerative colitis (UC) patients after quality control in the UK Biobank. Based on prior literature and biological plausibility , we tested 38 candidate environmental variables for association with CD, UC, and overall IBD using Cox proportional hazard regressions. We also tested whether these variables interacted with polygenic risk in predicting disease, following up significant (FDR < 0.05) results with tests for SNP-environment associations. We performed robustness analyses on all significant results. As in previous reports, appendectomy protected against UC, smoking (both current and previous) elevated risk for CD, current smoking protected against UC, and previous smoking imparted a risk for UC. Childhood antibiotic use associated with IBD, as did sun exposure during the winter. Socioeconomic deprivation was conferred a risk for IBD, CD, and UC. We uncovered negative interactions between polygenic risk and previous oral contraceptive use for IBD and UC. Polygenic risk also interacted negatively with previous smoking in predicting UC. There were no individually significant SNP-environment interactions. Thus, for a limited set of environmental variables, there was strong evidence of association with IBD diagnosis in the UK Biobank, and interaction with polygenic risk was minimal.

## Introduction

Inflammatory bowel disease (IBD) is a group of chronic, relapsing, debilitating conditions, primarily comprising two diseases: Crohn’s disease (CD) and ulcerative colitis (UC). Both are thought to arise from an inappropriate mucosal immune response to gut commensals. However, CD is characterized by transmural inflammation along the entire digestive tract whereas UC is dominated by mucosal ulcers in the colon and rectum^1^. IBD prevalence is highest in North America and Western Europe (0.3%), but its rising prevalence in other countries renders it a serious healthcare burden worldwide^2^.

IBD remains a difficult disease to treat because its pathogenesis is not well understood. The disease is induced by a combination of genetic and environmental factors, but the finer details remain largely elusive, especially with regards to the non-genetic risks. Genome-wide association studies (GWASs)^3,4^ have now identified hundreds of variants that associate with IBD, but our knowledge of the environmental factors and gene-environment (GxE) interactions that contribute to the disease remains limited. Previous studies^5,6^ have noted dozens of environmental factors that may influence risk for IBD, but these studies do not always reach the same conclusions, in part because of the practical challenges associated with achieving sufficient statistical power, minimizing bias, and controlling for confounding factors. Moreover, very few studies have probed the genetic-environment interactions that associate with IBD, whether it is interactions involving polygenic risk or single nucleotide polymorphisms (SNPs)^7,8^.

To better understand the environmental factors and GxE interactions for IBD, we performed a large cohort study in the UK Biobank, a repository containing genotype and phenotype information for approximately 0.5 million individuals in the UK. The UK Biobank’s cohort design offered the opportunity to study the epidemiology of IBD with less selection bias than typical case–control studies, which are more prevalent in the literature. We looked for environmental associations that predicted IBD independently of known genetic risk as well as any polygenic risk-environment interactions. Our observational findings can serve as the starting point for future experimental work on the role of these variables in IBD pathogenesis.

## Materials and methods

We used data from the UK Biobank, a “prospective cohort study with deep genetic and phenotypic data collected on approximately 500,000 individuals from across the United Kingdom, aged between 40 and 69 at recruitment.”^9^ UK Biobank carried out all data collection and methods in accordance with health research regulations in the United Kingdom. The UK Biobank study was approved by the North West—Haydock Research Ethics Committee in the United Kingdom under NHS Research Ethics Committee number 16/NW/0274. Informed consent was obtained during UK Biobank data collection. Enrollment of participants for long-term follow-up took place between 2006 and 2010; the data used in this study was retrieved in 2019. The phenotypic data was collected from a variety of sources, including in-person surveys and interviews conducted at enrollment on a range of topics including past diagnoses, periodically emailed dietary recall questionnaires, and hospital episode statistics (HES) records detailing past diagnoses and surgical operations.

We performed quality control on the dataset to ensure that our cohort was genetically homogenous and non-related. We excluded individuals who did not have British white ancestry (as in previous studies^10^, British white ancestry encompassed those who both identified as “white-unspecified,” “white-British,” or “Irish” and were within 7 standard deviations of the mean for the first 6 genetic principal component measures) because we did not expect homogeneity of effects across ancestral groups and because the number of non-white patients was too small in the UK Biobank to have sufficient power to study. We also removed individuals who had close kin in the cohort based on estimated kinship coefficients. Specifically, participants who were related to multiple other participants to the first, second, or third degree were excluded. From each remaining related pair, a random participant was excluded and a random one was kept. Cryptic relatedness was determined using the package KING, with settings designed to exclude third-degree relatives and closer, as described previously^9^. In addition, we excluded those with sex chromosome aneuploidies from analysis. 364,908 individuals from the original 488,377 passed quality control, including 168,992 males and 195,916 females with a median age of 68.

**Figure 1:**
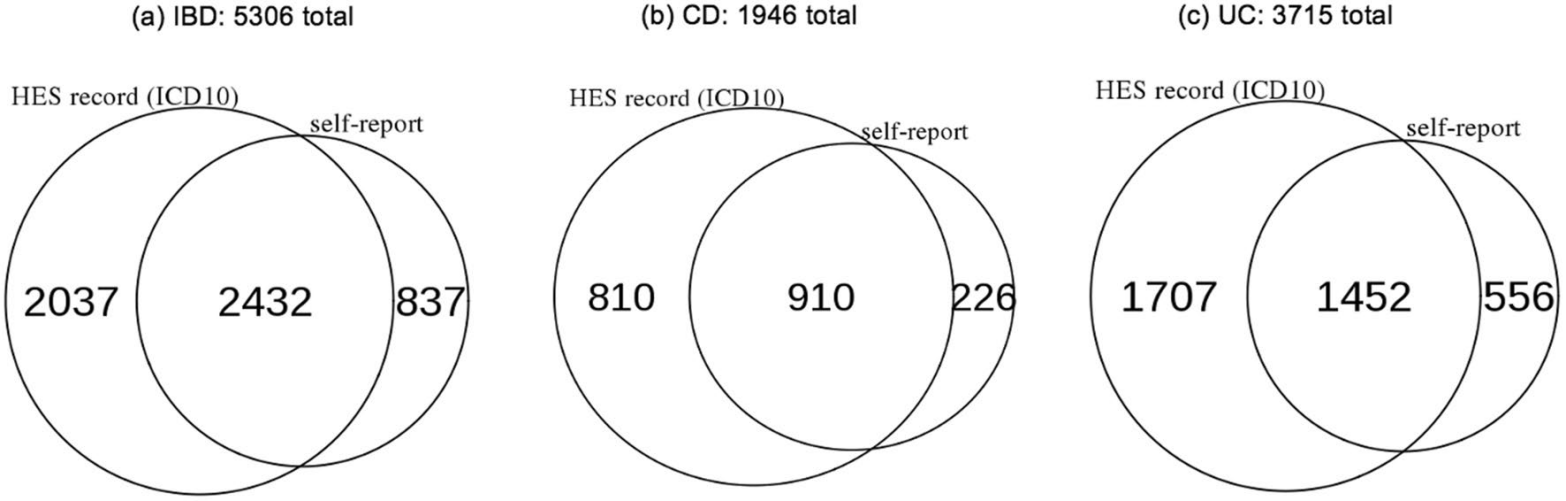
Number of IBD cases in the UK Biobank based on method of identification. ICD10-coded diagnoses were recorded in hospital episode statistics (HES) while self-reported conditions were gathered by survey at recruitment.

We identified 5306 individuals who either self-reported a previous diagnosis of IBD or had IBD coded (ICD-10) in their hospital episode statistics (HES). 1946 had CD and 3715 had UC (Fig. 1). There was considerable overlap between those who self-reported IBD and those with IBD in the HES records (46% of IBD cases were identified through both routes), but there were also discrepancies. These were likely due to misremembered diagnoses, patients seeking medical care outside the NHS, or the lack of complete HES coverage prior to 1997. In fact, 18.5% (67,423 individuals) in the cohort did not have any HES data at all.

**Figure 2:**
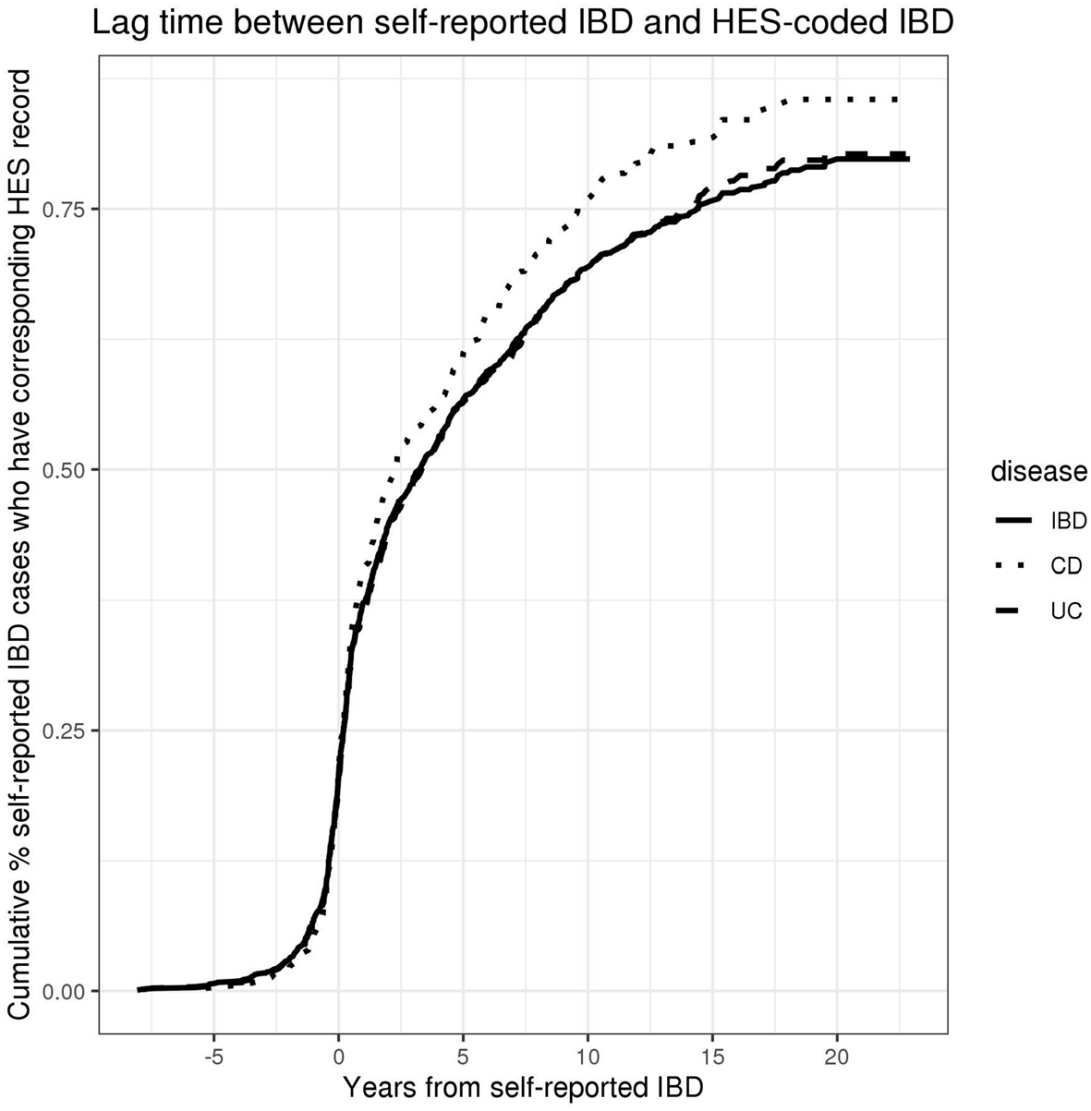
Lag time between self-reported IBD and HES-coded IBD for patients self-reporting IBD after 1997, measured by number of years from earliest recollection of first diagnosis to first instance of relevant ICD10-code in HES records.

To properly characterize the overlap in self-reported versus hospital record diagnoses, we looked at those individuals with an IBD diagnosis after 1997, which is when HES data became available across the United Kingdom. Of the 1208 individuals who self-reported an IBD diagnosis after 1997, 912 (75.5%) also had an HES-coded IBD diagnosis. This high but incomplete overlap between self-reported and HES-coded diagnoses in the UK Biobank has been noted for other phenotypes, and genetic evidence suggests that these phenotyping methods identify comparable sets of cases^11^. We also compared timings of diagnoses for those with self-reported IBD after 1997 and found that earliest HES-coded diagnosis was usually either in the same year (27.0%) or later (60.1%) than the recalled date of diagnosis, but that there was a highly variable lag time between recalled date of diagnosis and date of first HES record (Fig. 2). In our analyses below, we therefore rely on HES records and self-reported diagnoses separately.

Of note, we do not include indeterminate colitis (ICD10 code: K52.3) in our definition of IBD. We also interpret every HES record as a definitive diagnosis, although we note that coding errors, diagnostic errors, and diagnostic changes can occur in IBD, which may affect the results of this study. We therefore carried out a sensitivity analysis in which we removed the 592 patients (Supplemental Table 2) with either conflicting CD and UC diagnoses or a HES record of indeterminate colitis.

Among IBD cases there were 2602 males and 2704 females with median age 69, which is nearly identical to the median age of the entire cohort, 68 (Supplemental Fig. 1). There was a sex difference in IBD prevalence (1.54% in males vs. 1.38% in females, *p* = 1.63e−04). Previous studies have indicated that this sex difference can be attributed to the increased prevalence of UC in older males, and this is indeed the case in the UK Biobank (1.12% in males vs. 0.927% in females, *p* = 2.85e−08)^12^.

### Genetic risk

Using 232 previously identified biallelic SNPs^3^ associated with IBD, CD, and UC, we calculated polygenic risk scores (PRSs) for each of the three disease phenotypes according to the following standard formula

1 $$PRS = \mathop \sum \limits_{SNP} \beta_{SNP} \,*\,g_{SNP}$$

where $\beta$ is effect size and *g* is genotype represented by 0, 1, or 2. We then tested whether our PRSs associated with their respective disease phenotypes in a logistic regression, with age, sex, genetic ancestry, and location of the UK Biobank assessment center as covariates (Eq. 2).

2 $${\text{log}}\left( {{\text{odds}}\,{\text{of}}\,{\text{ IBD}}} \right) = \beta_{0} + \beta_{1} PRS + \mathop \sum \limits_{i} \beta_{i} {\text{covariate}}_{i}$$

### Environmental associations and GxE interactions

We used Cox proportional hazards regressions to model the risk imparted by various environmental variables in right-censored survival analyses where the event of interest was IBD diagnosis as noted in self-report surveys (using the participant’s recalled year of diagnosis) or HES records (taking the earliest hospital episode as the date of diagnosis). To minimize confounding and to control for demographic factors and known genetic risk, we typically included the following covariates in our models: PRS, age, sex, 10 genetic principal components (ancestry), and UK Biobank assessment center location (Eq. 3). There were exceptions, however (also refer to Table 1): When testing the effects of 24-h dietary recall variables, we added daily caloric intake as an extra covariate to control for total consumption. When testing for geographic variables—namely, socioeconomic status, latitude at birth, latitude at recruitment, and sun exposure during the summer and winter—we removed the location of the assessment center from the usual list of covariates to avoid collinearity. This information is summarized in Table 1.

**Table 1:** Characteristics of environmental variables investigated. PRS = polygenic risk score. OCT = oral contraceptive therapy. HRT = hormone replacement therapy.

Environmental variable	# of CD cases/# of participants in analysis	# of UC cases/# of participants in analysis	Prospective versus retrospective	Notes on variable definitions	Covariates used in regression
**Diet variables**					
Diet pattern—4 variables (frequency per week): red meat, processed meat, fresh fruit, alcohol	~ 400/ ~ 355,000 (differs slightly for each variable)	~ 910/ ~ 355,000 (differs slightly for each variable)	Prospective		PRS, age, sex, 10 genetic principal components (ancestry), UK Biobank assessment center location
24-h dietary recall—17 variables (amount consumed daily based on 24 h recall): fiber, fat, polyunsaturated fats, saturated fats, sugar, alcohol, iron, calcium, potassium, magnesium, protein, vitamin B6, folate, vitamin B12, vitamin C, vitamin D, vitamin E	10/18,291	31/18,291	Prospective	We did not include intake of vitamin supplements in our analysis because supplemental intake is not quantified in the UK Biobank	PRS, age, sex, 10 genetic principal components (ancestry), UK Biobank assessment center location, daily caloric intake
**Geographic variables**					
Socioeconomic deprivation (Index of Multiple Deprivation 2010)	439/353,075	961/353,375	Prospective		PRS, age, sex, 10 genetic principal components (ancestry)
Sun exposure during the summer (hours spent outdoors on a typical day)	450/361,895	990/362,192	Prospective		
Sun exposure during the winter (hours spent outdoors on a typical day)	450/361,895	990/362,192	Prospective		
Latitude at recruitment	446/358,812	980/358,812	Prospective		
Latitude at birth	1088/345,732	1915/345,732	Retrospective		
**Perinatal variables**					
Cesarean section	354/124,664	680/124,664	Retrospective		PRS, age, sex, 10 genetic principal components (ancestry), UK Biobank assessment center location
Breastfed as baby	1134/364,796	1998/364,796	Retrospective		
Maternal smoking around birth	1108/359,405	1972/359,405	Retrospective		
**Drugs and surgeries**					
Appendectomy	1946/364,898	3714/364,898	Retrospective		PRS, age, sex, 10 genetic principal components (ancestry), UK Biobank assessment center location
Prolonged exposure to antibiotics during childhood (surveyed)	288/119,936	604/119,927	Retrospective		
Regular NSAID use	451/361,849	989/361,849	Prospective	Includes aspirin. Participants were classed as “regular users” if they used NSAIDs 4 or more times a week for the past for weeks at time of survey	
Smoking (current use)	1684/310,960	3154/310,960	Retrospective (time-varying)	Participants who provided ages for starting (or stopping) smoking which did not fall within 5 years of each other were removed. Those who did not smoke for longer than a year were excluded	
Smoking (previous use)	1684/310,960	3154/310,960	Retrospective (time-varying)		
Oral contraceptive therapy (current use)	942/175,001	1627/175,001	Retrospective (time-varying)	Participants who provided ages for starting (or stopping) OCT which did not fall within 5 years of each other were excluded. Those who did not use OCT for longer than a year were excluded	
Oral contraceptive therapy (previous use)	942/175,001	1627/175,001	Retrospective (time-varying)		
Hormone replacement therapy (current use)	942/175,001	1627/1,750,001	Retrospective (time-varying)	Participants who provided ages for starting (or stopping) HRT which did not fall within 5 years of each other were excluded. Those who did not use HRT for longer than a year were excluded	
Hormone replacement therapy (previous use)	942/175,001	1627/1,750,001	Retrospective (time-varying)		

We corrected for multiple hypothesis testing using Benjamini–Hochberg adjustments to control false discovery rate at FDR < 0.05 within each disease subtype. All significant results were checked graphically; poorly fit models and data which did not visually conform to the proportional hazards assumption were discarded (only models for hormone replacement therapy were discarded).

Based on previous findings and biological plausibility^5,6,13,14^, we chose 38 environmental variables to test for association with IBD (Table 1). For each environmental variable, any individual with incomplete or illogical data (e.g., started smoking before quitting smoking) was excluded from analysis. All but three variables were assessed through a single recall event which was collected via touchscreen survey and verbal interview at recruitment; for the vast majority of participants (~ 95%), there was no further follow-up for these variables. For the small minority of participants with follow-up responses to survey questions, the averaged value of their responses and the date of the initial survey were used in the analysis. The three exceptions to this method of data collection were appendectomy, socioeconomic status, and 24-h diet recall. Appendectomy was both assessed by recall at enrollment and gathered from HES records, whichever occurred earlier. Socioeconomic deprivation was determined by matching participant zip codes at recruitment against the UK government’s Index of Multiple Deprivation (IMD) from 2010. And in contrast to all other variables, 24-h diet recall was assessed through multiple recall events. Participants were sent questionnaires five times between 2009 and 2011 at approximately 4–6 month intervals asking them to recall what they consumed in the past 24 h. For accuracy, we excluded those who did not respond to at least three of the five questionnaires.

3 $${\text{log}}\left( {{\text{hazard }}\,{\text{ratio }}\,{\text{for}}\,{\text{IBD}}} \right) = \beta_{1} PRS + \beta_{2} E + \mathop \sum \limits_{i} \beta_{i} {\text{covariate}}_{i}$$

We also asked whether any of the environmental variables interacted with the PRS by adding an interaction term to the Cox model (Eq. 4).

4 $${\text{log}}\left( {{\text{hazard}}\,{\text{ratio}}\,{\text{for}}\,{\text{IBD}}} \right) = \beta_{1} PRS + \beta_{2} E + \beta_{3} PRS*E + \mathop \sum \limits_{i} \beta_{i} {\text{covariate}}_{i}$$

**Figure 3:**
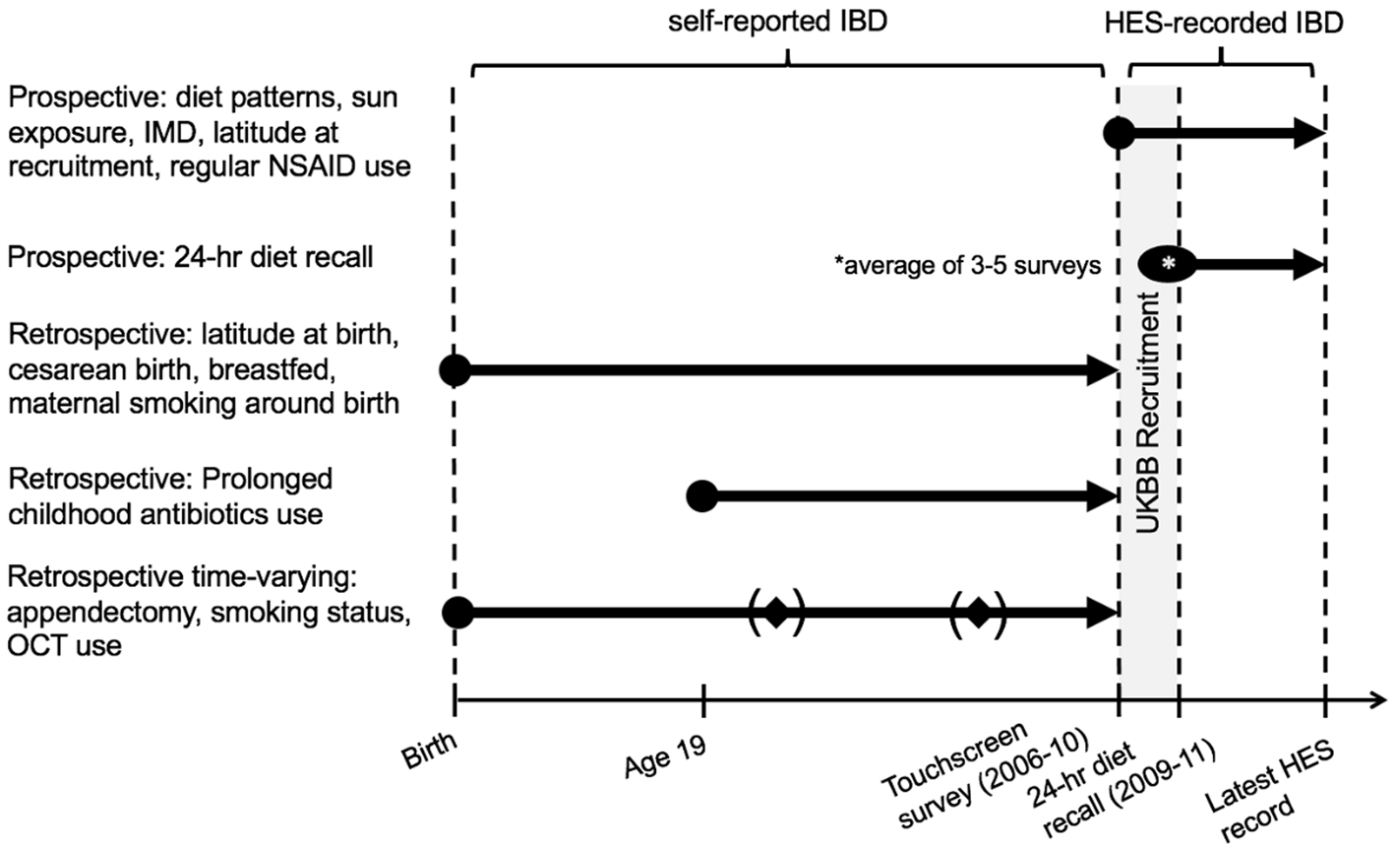
Schematic of the primary analyses we carried out for each variable. All variables were measured at a single time point except 24-h diet recall, which represented the average value from 3 to 5 surveys taken at multiple time points. IBD = inflammatory bowel disease, IMD = Index of Multiple Deprivation (a measure of socioeconomic status), OCT = oral contraceptive therapy.

We chose to separate our analysis of self-reported IBD and HES-coded IBD because we found a variable lag time between self-reported IBD after 1997 (when HES data became available) and HES-recorded diagnoses in the UK Biobank (Fig. 2). That is, in each survival analysis and regression model we used either self-reported IBD or HES-recorded IBD depending on whether the analysis was prospective (in which case we relied on HES data) or retrospective (in which case we used self-reported data). Our approach is displayed in Fig. 3.

Prospective analyses were conducted for variables that dealt with environmental exposures around the time of enrollment. Data on diet patterns, socioeconomic status, summer and winter sun exposure, latitude at recruitment, and regular non-steroidal anti-inflammatory drug (NSAID) use were all collected via a touchscreen survey at the time of enrollment. Their association with IBD was tested in prospective analyses that relied on HES data for diagnoses of IBD after enrollment. These analyses began at the time of enrollment and proceeded until either a diagnosis of IBD was made or the date of the patient’s most recent HES record.

Prospective analyses were also carried out for the 24-h dietary recall variables, which are distinct from the dietary patterns data collected via the touchscreen survey. These 24-h dietary recall variables were collected through a series of 5 questionnaires sent to participants over the period 2009 to 2011. These prospective analyses began at the time of the first questionnaire completed and proceeded until either a diagnosis of IBD was made through HES or the date of the patient’s most recent HES record.

Retrospective analyses were carried out for perinatal and childhood variables–namely, birth by cesarean section, being breastfed, maternal smoking around birth, and prolonged childhood exposure to antibiotics. Analyses for perinatal variables began at birth and proceeded until a self-reported diagnosis of IBD (since HES records were not available during these years) or the date of enrollment into the UK Biobank (since this is when self-reported diagnoses were collected via the touchscreen survey). The analysis of childhood exposure to antibiotics begans instead at age 19–the definition of “childhood” for this variable in the UK Biobank–but otherwise was the same.

Finally, time-varying retrospective analyses were carried out for lifespan variables whose statuses could change across a participant’s life. These included appendectomy, smoking status, hormone replacement therapy use, and oral contraceptive use. These were the only variables which underwent a time-varying analysis. The analyses began at birth and proceeded with time-varying changes to a participant’s exposure status until either self-reported IBD or the date of enrollment.

Because of the uncertainty around the dating of IBD diagnosis in the UK Biobank, and because recorded IBD diagnosis typically lags behind real diagnosis of disease, we performed robustness analyses around the point of truncation whereby individuals suspected of having IBD before the truncation point were removed even if their recorded date of diagnosis fell after that time. We did this through one of three ways: (1) additionally removing all IBD cases diagnosed within 2 years after the survey, (2) additionally removing all those who had surgeries (excisions into small intestine, colon, and rectum) commonly performed in IBD patients before the survey, and (3) additionally removing all those who had either IBD-related surgeries or endoscopies before the survey (relevant OPSC-4 codes in Supp. Table 1). Participants ruled out from analysis based on their histories of surgeries and endoscopies were enriched for future IBD (for surgery only: 4 out of 11,918 vs. 68 out of 329,783, OR = 1.63, *p* = 0.32 by Fisher’s exact test; for both surgery and scope: 4 out of 6393 vs. 68 out of 355,312, OR = 3.27, *p* = 0.039), indicating that our methods did indeed target potential IBD cases who simply had not been identified as such before truncation. These removals may introduce new biases into the analysis, so we only used these analyses to test the robustness of the statistically significant results in the original data and not to draw new conclusions.

For analyses without left truncation, we performed a different robustness analysis whereby we removed individuals whose date of IBD diagnosis fell within 2 years after a change in environmental status—for instance, getting an appendectomy or quitting smoking.

Lastly, to assess the possible role of recall errors, we conducted sensitivity analyses for the lifespan variables—i.e., appendectomy, smoking, hormone replacement therapy, and oral contraceptive therapy (OCT)—using an alternative prospective analysis. Follow-up began at enrollment and proceeded until either a diagnosis of IBD on HES records or the date of the last HES record. The statuses of these variables were fixed at baseline for the vast majority of individuals since post-enrollment data for these variables were gathered by the UK Biobank only in a minority (~ 5%) of participants. The sensitivity results for current OCT use were discarded because only 2 active OCT users were diagnosed with IBD after enrollment.

## Ethical considerations

All patient data was collected by the UK Biobank (NHS REC number 16/NW/0274), accessed under approved application number 11805, and analyzed on secure university servers. UK Biobank carried out all data collection and methods in accordance with health research regulations in the United Kingdom. The UK Biobank study was approved by the North West—Haydock Research Ethics Committee in the United Kingdom. Informed consent was obtained during UK Biobank data collection.

## Results

**Figure 4:**
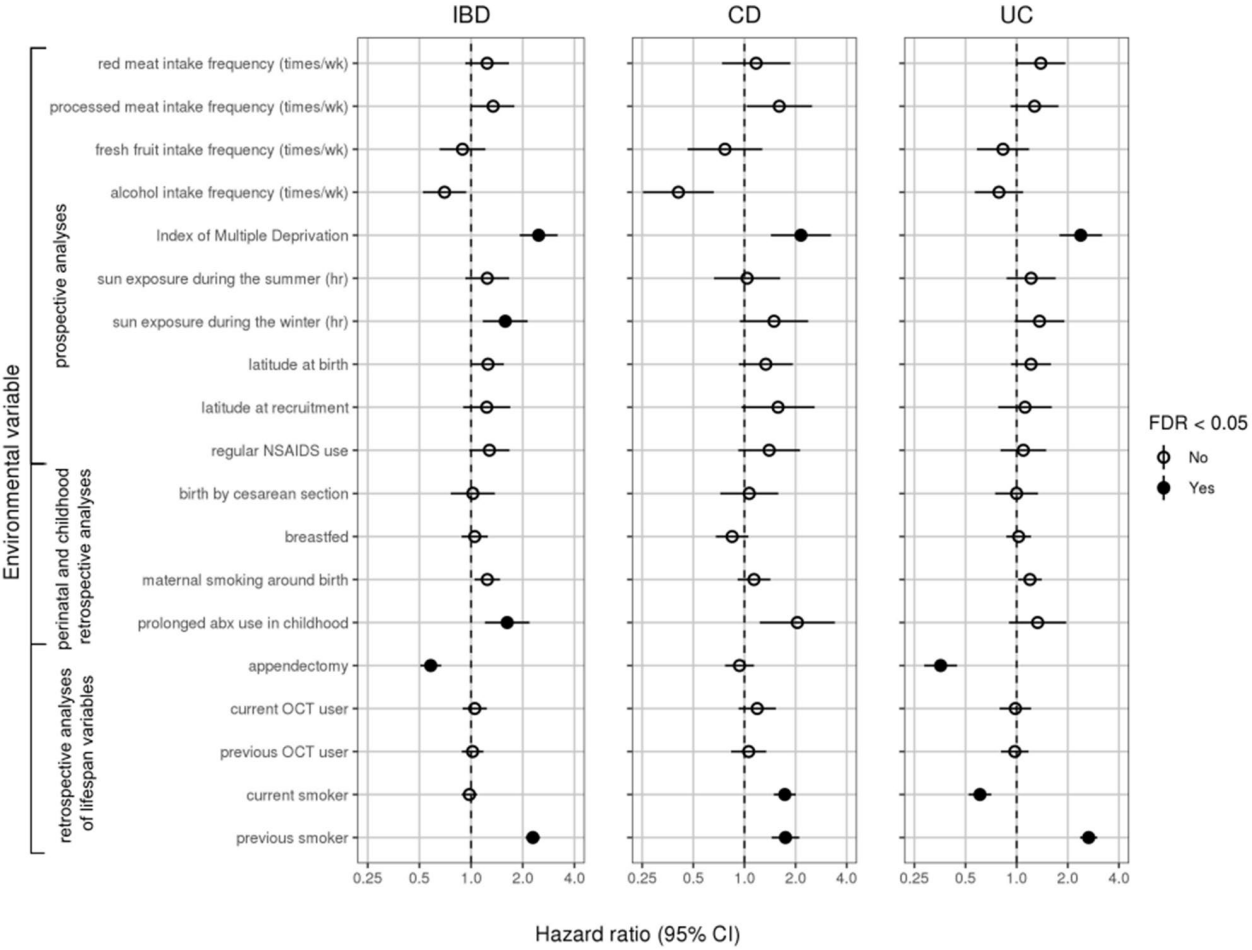
Forest plot of hazard ratios (dots) and 95% confidence intervals (lines) obtained from Cox regressions. Hazard ratios were adjusted for other covariates, including polygenic risk. Statistically significant results (FDR < 0.05) represented by filled circles. For continuous variables, hazard ratios are given per standard deviation of the variable. For binary variables, raw hazard ratios are given. Results for 24-h dietary variables are shown in Supplemental Fig. 3. Results for hormone replacement therapy not displayed because they did not meet the proportional hazards assumption.

**Figure 5:**
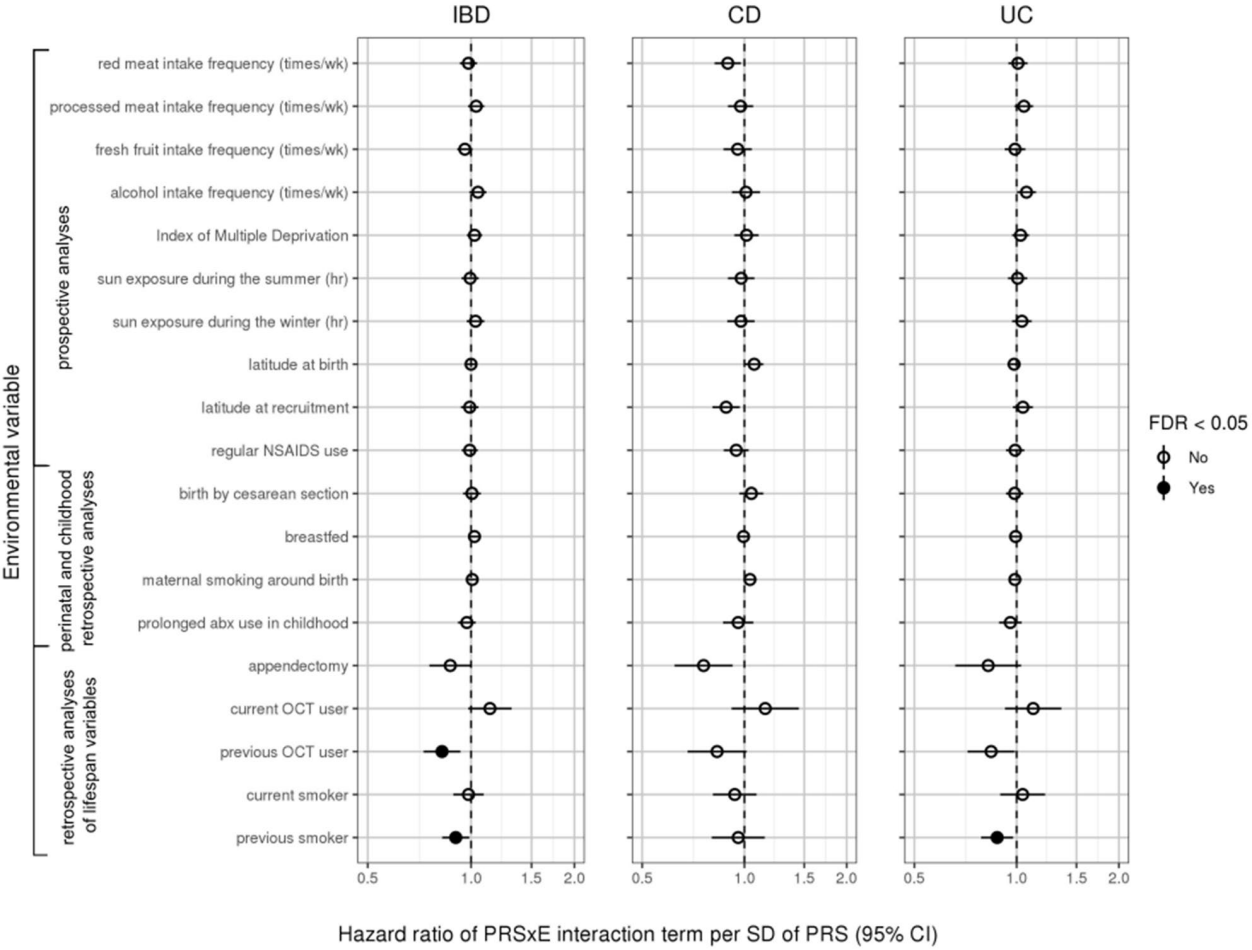
Forest plot of hazard ratios (dots) and 95% confidence intervals (lines) for PRSxE interactions obtained from Cox regressions. Hazard ratios were adjusted for other covariates, including polygenic risk. Statistically significant results (FDR < 0.05) represented by filled circles. x-axis truncated at 2. For continuous variables, hazard ratios are given per standard deviation of the variable per standard deviation of PRS. For binary variables, hazard ratios are given per standard deviation of PRS. Results for 24-h dietary variables are shown in Supplemental Fig. 4. Results for hormone replacement therapy not displayed because they did not meet the proportional hazards assumption.

**Figure 6:**
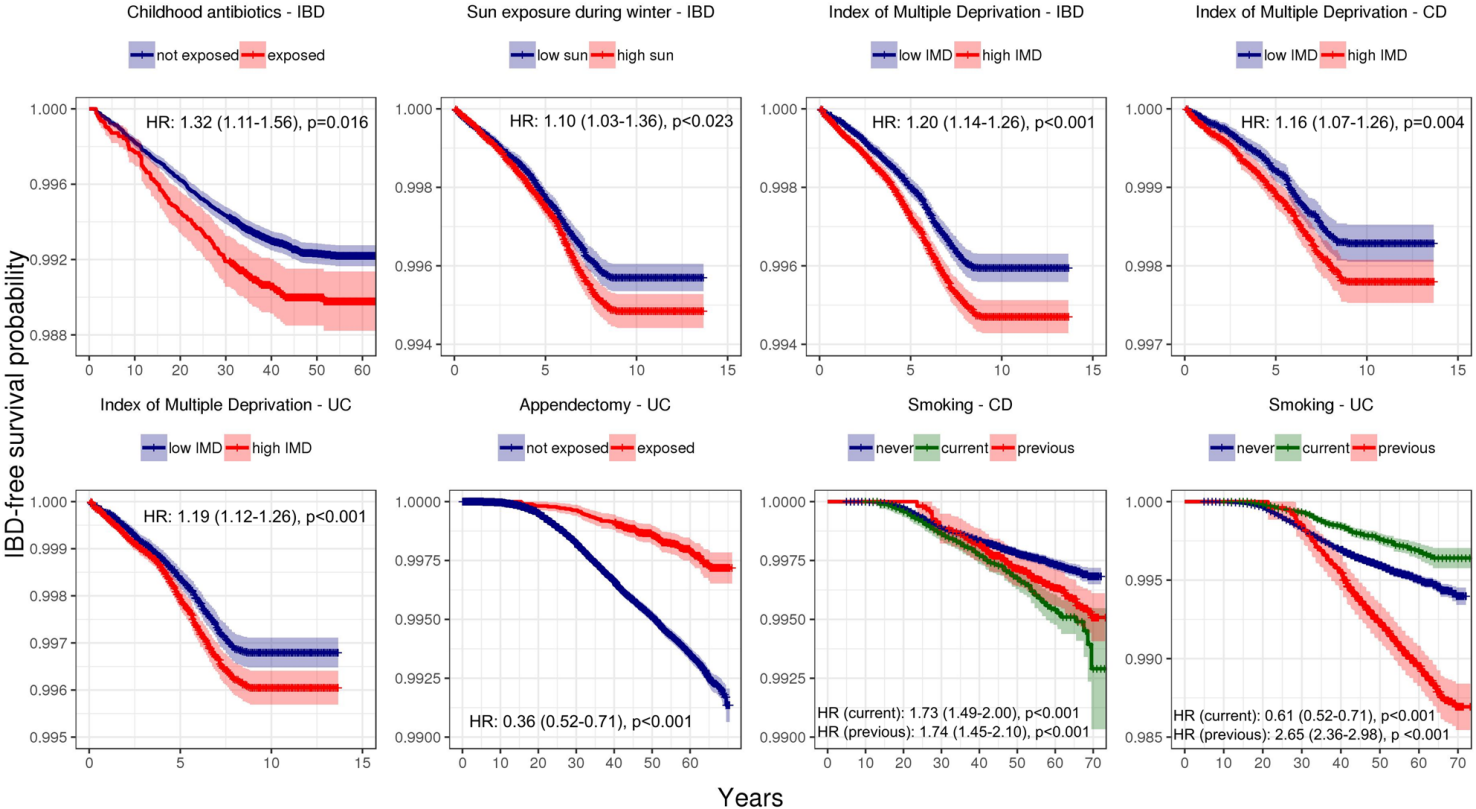
Kaplan–Meier curves for the statistically significant (FDR < 0.05 in Cox regressions conditional on polygenic risk and other covariates) environmental variables with IBD diagnosis as the event. Shading indicates 95% confidence interval. For IMD, hazard ratios are given per standard deviation of the variable. IMD = Index of Multiple Deprivation.

**Figure 7:**
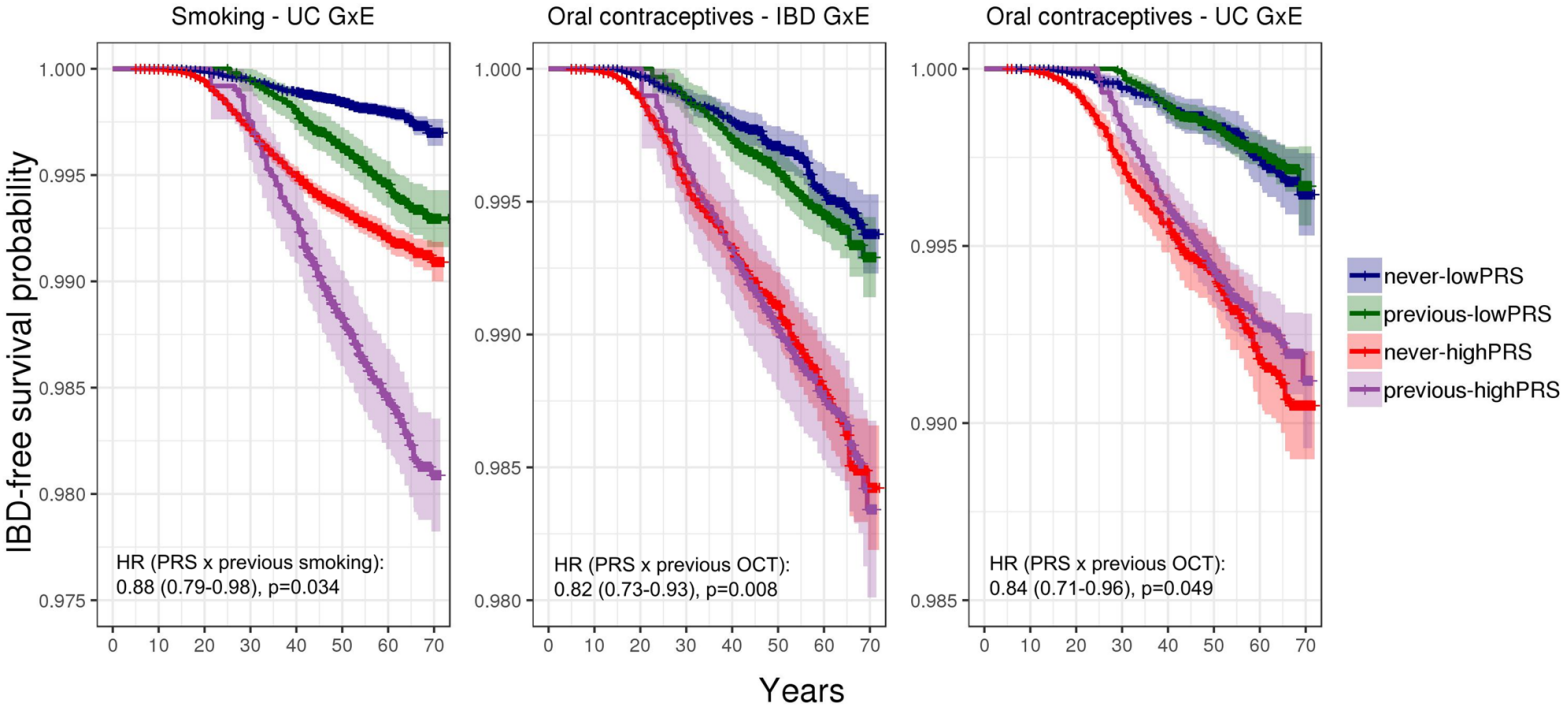
Kaplan–Meier curves for variables with statistically significant PRSxE interactions (FDR < 0.05 in Cox regressions conditional on polygenic risk and other covariates), with IBD diagnosis as the event. “Never” indicates participants never exposed to variable, “previous” refers to participants who started and subsequently stopped exposure, “lowPRS” refers to polygenic risk below median, “highPRS” refers to polygenic risk above median. Curves of current users are omitted to emphasize the interactive effect. OCT = oral contraceptive therapy.

Environmental associations are shown as forest plots in Fig. 4 (all variables except the 24-h dietary recall variables) and Supplemental Fig. 6 (24-h dietary recall variables) as well as tabulated in Supplemental Table 3 (all variables). Gene-environment interaction results are shown in Fig. 5 (all variables except the 24-h dietary recall variables) and Supplemental Fig. 7 (24-h dietary recall variables). Survival curves of statistically significant (FDR < 0.05) marginal associations and gene-environment interactions are shown in Figs. 6 and 7, respectively.

### Genetic risk

The PRSs constructed for IBD, CD, and UC all associated strongly with their respective diseases in multivariable logistic models, with the coefficient adjusted per standard deviation of the PRS (95% CI for IBD: 1.29–2.16, *p* < 0.001; 95% CI for CD: 1.65–1.80, *p* < 0.001; 95% CI for UC: 1.76–1.88, *p* < 0.001; see Supplemental Fig. 2 and Supplemental Table 4).

### Diet

The UK Biobank assesses dietary habits in two ways: (1) by surveying participants on how frequently they consume certain food categories, e.g. beef or pork, and (2) by using mail-in questionnaires asking patients periodically to recall everything they had consumed in the past 24 h, from which daily nutritional intake is inferred. We did not find strong evidence for dietary associations with IBD diagnosis in the current UK Biobank data (Fig. 4, Supplemental Figure 3).

### Geography and socioeconomic status

In prospective analyses, socioeconomic deprivation as measured by the Index of Multiple Deprivation (IMD) increased risk for CD (95% confidence interval for hazard ratio per standard deviation IMD: 1.07–1.26, adjusted *p* = 0.004), UC (95% CI 1.12–1.26 per standard deviation IMD, adjusted *p* < 0.001), and IBD (95% CI 1.14–1.26 per standard deviation IMD, adjusted *p* < 0.001). These results held when we applied a 2-year buffer for diagnosis, though they mostly did not hold when we truncated those with previous IBD-related surgeries and/or endoscopies (Supplemental Figure 5). We did not find a significant association between latitude and IBD within the UK, whether the latitude was measured at birth (95% CI 1.00–1.09 for IBD, 0.98–1.14 for CD, 0.98–1.10 for UC per standard deviation in latitude) or at recruitment (95% CI 0.98–1.11 for IBD, 0.99–1.21 for CD, 0.95–1.10 for UC per standard deviation in latitude). We also found no significant associations between sun exposure and IBD within the UK other than a positive association between hours of sun exposure during the winter and overall IBD (95% CI 1.03–1.16 per standard deviation in sun exposure, adjusted *p* = 0.023). However, this result was also the only significant finding in this study that did not replicate in any of the robustness tests, indicating it may have been a false positive.

### Perinatal factors

None of the perinatal factors associated significantly with IBD.

### Drugs and surgeries

We found no significant associations for self-reported regular NSAIDs use (95% CI 0.99–1.26 for IBD, 1.00–1.49 for CD, 0.91–1.20 for UC) or oral contraceptive therapy (current OCT use: 95% CI 0.89–1.24 for IBD, 0.92–1.53 for CD, 0.79–1.22 for UC; previous OCT use: 95% CI 0.88–1.18 for IBD, 0.84–1.34 for CD, 0.81–1.18 for UC). Self-reported repeated use of antibiotics during childhood (defined as < 20 years old) was linked to increased risk for overall IBD (95% CI 1.11–1.56, adjusted *p* = 0.016) but not CD and UC individually (95% CI 1.13–2.01 for CD, 0.94–1.47 for UC), perhaps due to insufficient power. This positive association was replicated in further robustness analyses in which we implemented a 2-year time buffer around truncation or eliminated those who had IBD-related surgeries before the date of truncation. The data for hormone replacement therapy (HRT) did not follow the Cox proportional hazards assumption due to a lack of HRT users, so no modeling was performed (data not shown).

We replicated previously uncovered effects for appendectomy and tobacco smoking^15–20^. Specifically, we found that appendectomies were protective against UC (95% CI 0.52–0.71, adjusted *p* < 0.001). However, we did not find that appendectomy conferred a risk for CD as others have previously (95% CI 0.77–1.14)^21,22^. Meanwhile, we found that tobacco smoking was a risk for CD (95% CI 1.49–2.00 for current smokers, adjusted *p* < 0.001, and 1.45–2.10 for previous smokers, adjusted *p* < 0.001), and it protected current smokers against UC (95% CI 0.52–0.71, adjusted *p* < 0.001) while conferring a risk for UC to previous smokers (95% CI 2.36–2.98, adjusted *p* < 0.001). These results were all replicated in robustness analyses.

### GxE interactions

We uncovered three significant interactions between the PRS and an environmental variable (Figs. 5 and 7). Specifically, we found that although previous OCT use did not exhibit a marginal association with IBD (95% CI 0.88–1.18), it elevated the risk for IBD and UC in individuals with lower polygenic risk but attenuated the risk for IBD and UC in those with higher polygenic risk (95% CI for hazard ratio of interaction term per standard deviation PRS: 0.73–0.93 for IBD, adjusted *p* = 0.008; 0.71–0.96 for UC, adjusted *p* = 0.049). Meanwhile, we found that the risk which previous smoking confers for UC is attenuated in individuals with high genetic risk (95% CI for hazard ratio of interaction term per standard deviation PRS: 0.79–0.98, adjusted *p* = 0.034). We probed each of these three PRS-environment interactions for individual SNP-environment interactions but did not find any significant results (data not shown). The SNPs were not in strong linkage disequilibrium (the three highest R^2^ values were 0.480, 0.209, and 0.184).

### Prospective modeling for lifespan variables

The prospective sensitivity analyses we conducted for the lifespan variables—i.e., appendectomy, smoking, OCT—produced environmental associations which generally agreed with the results obtained in the primary retrospective analysis (Supplemental Fig. 7). We found that smoking continued to be a risk for CD (95% CI 1.43–2.33 for current smokers and 1.40–1.96 for previous smokers) and a risk for UC after quitting (95% CI 1.64–2.11). Active smoking was not significantly associated with UC (95% CI 0.96–1.48), but the confidence interval overlapped with our main result. Appendectomy was again protective against UC (95% CI 0.58–0.84) and IBD (95% CI 0.71–0.96) but not CD (95% CI 0.93–1.41). The PRS-environment interactions obtained from the prospective analyses overlapped with the confidence intervals of the main results, but they were not statistically significant (95% CI for hazard ratio of interaction term per standard deviation PRS: 0.83–1.46 for previous OCT and IBD, 0.82–1.61 for previous OCT use and UC, 0.92–1.18 for previous smoking and UC.)

### Further sensitivity analyses

In the above analyses, we carried out multiple testing corrections separately by Benjamini–Hochberg within the 36 results for each disease. To test how robust our results were to the multiple testing approach, we also carried out the Benjamini–Hochberg procedure across all 108 tests across all diseases simultaneously. 10 out of 12 results remained significant. One additional result became significant (childhood antibiotics and CD), and two became non-significant (OCT use and IBD, appendectomy and IBD).

We also conducted a sensitivity analysis in which we removed 592 unique patients who either had conflicting histories of CD and UC (on self-report or HES record) or had a HES record of indeterminate colitis. Removing these samples had only minor impacts on the results (Supplemental Fig. 8 and Supplemental Table 5).

## Discussion

Our large cohort analysis replicated many previously established IBD risk factors. Specifically, we found that appendectomy protects against UC, both current and previous smoking confer risk for CD, active smoking protects against UC, and previous smoking confers risk for UC. We replicated the majority of these findings across a range of sensitivity analyses, controlling for delays between onset and diagnosis, and in prospective analyses. One exception was the effect of active smoking on UC, which was not statistically significant. This could have resulted from the paucity of additional data on smoking status after enrollment or possible age-related modifiers of smoking’s effects. Dietary variables such as pork^23^ and fiber^24^ have also been linked to IBD, but we did not find significant dietary associations, most likely due to significantly diminished statistical power for 24 h-recall dietary variables (only 18,291 participants met our inclusion criteria). We did not replicate previously noted associations between appendectomy and CD in our retrospective analyses (our confidence intervals did not overlap a previous meta-analysis^22^), though our prospective sensitivity analyses showed greater overlap between our estimates and previous estimates, suggesting possible recall bias in the retrospective analysis.

The extensive phenotyping in the UK Biobank allowed us to investigate environmental factors which have previously been understudied. It is known that IBD patients of lower socioeconomic status have worse outcomes^25^, but few studies have tested the influence of socioeconomic status on IBD diagnosis. In the UK Biobank, socioeconomic deprivation was a risk for both CD and UC diagnosis, and these results were replicated in a robustness test. Antibiotics use has been shown to be a risk factor for pediatric IBD^26^, but we found that recalled recurrent antibiotics use during childhood was also linked to adult-onset IBD. Only one study we know of has investigated the relationship between maternal smoking and IBD, and we replicated its finding of no association^27^. Previous studies have also reported that IBD risk increases with latitude, but most of these studies span large differences in geography^28–31^. Studies across smaller territories have found less compelling results, and likewise we did not find such an association in the UK^32–35^. Lastly, sun exposure has been suggested to be protective for IBD^36^. We found an association between winter sun exposure and IBD, but this might have been a false positive as it did not pass our robustness tests. Further investigations are needed for these variables.

We also studied factors which have disputed results in the literature. Previous reports on perinatal factors have largely equivocated on their effects on IBD, though meta-analyses have suggested that breastfeeding is protective for IBD^37,38^ and cesarean sections confer a risk for CD^39,40^. We found no significant association between these two factors and IBD, though our confidence intervals overlapped previous estimates (Supp. Table 2). Finally, some studies report that OCT elevates risk for CD and UC among smokers specifically, while others find no association^41–43^. We found no cohort-wide associations for OCT use, but additional studies are needed, especially those which take dosage into consideration.

Although there was no consistent effect of previous OCT use on IBD or UC risk across our study, we observed varying effects of OCT on individual risk depending on genetic risk profile in our retrospective analysis. Biologically, estrogen is known to play important roles in cellular and humoral immunity^44^, colonic barrier function^45^, and microvascular thrombogenesis and secondary gut ischemia^46^. Replicating this GxE finding and understanding why OCT amplifies IBD or UC risk for some individuals but attenuates it in others, depending on genotype, could shed light on the role of hormone pathways in IBD pathogenesis.

The role of smoking on IBD is paradoxical, with current smoking protecting against UC but predisposing to CD, whereas previous smoking is risk for both. While the paradoxical relationships between smoking and IBD are well established^19^, the biology underpinning these different risk profiles has been long debated, and multiple intestinal pro-inflammatory and anti-inflammatory pathways impacted by smoking^47^. Our GxE analysis adds a further layer of complexity to this story, by showing that previous smoking (but not current smoking) has a larger effect on UC risk for those at lower genetic risk of UC. Smoking is known to have both short- and long-term effects on the immune system^48^, and if we hypothesize that the impacts on IBD can be separated into short-term effects (current smokers, risk for CD, protective for UC) and longer-term damage (previous smokers, risk for both), then our data suggests the long-term pathways specifically overlap and interact with genetic risk pathways. For both OCT and smoking, larger sample sizes in future studies will allow us to segregate the PRS interaction into individual loci, allowing us to identify the specific genetic risk pathways that are more susceptible to hormonal or smoking-induced dysregulation.

This study had three important advantages. One was that it was done in a large cohort, which allowed us to reduce sampling error and achieve greater statistical power than many previous studies. Second, the inclusion of covariates in our regression models to control for known genetic risk, genetic ancestry, and various pre-existing demographic and geographic factors, reducing the impact of confounding. Finally, by conducting survival analyses in a cohort, we were better able to consider the timing of environmental exposures relative to IBD and focus on how pre-disease exposures influence risk for IBD.

Our study faces several important limitations. It is impossible to rule out recall inaccuracies fully, though self-reported diseases in the UK Biobank have been cross-validated with HES records in genome-wide association studies^11^. Similarly, coding errors in medical records or changes in diagnosis may introduce biases. While our sensitivity analyses indicate that these forms of diagnostic errors did not substantially distort our findings for environmental associations; the gene-environment interactions in our prospective sensitivity analyses were no longer statistically significant, though the confidence intervals still overlapped with the main results. This loss of significance may reflect the reduced statistical power, age-related effects, or differences between self-reported and HES-coded IBD. Next, we cannot draw causal conclusions from our findings, and although we took care to study pre-disease environmental exposures, we cannot rule out the possibility of reverse causality due to diagnostic delay^49^. Lastly, the associations we found are not necessarily generalizable to populations outside white British individuals. The epidemiology of non-white IBD patients is particularly understudied, and this disparity can only be addressed in cohorts with sufficiently large non-white participants. Hence, studies in data sets from other populations would be very illuminating, such as the China Kadoorie Biobank, the Million Veterans Project, and the Genetic, Environmental, Microbial (“GEM”) Project by Crohn’s and Colitis Canada.

## Conclusion

Using survival analyses, we searched for environmental and GxE associations with IBD diagnosis in a large UK cohort. For a limited set of environmental variables there is strong evidence of association with IBD diagnosis in the UK Biobank, and some show evidence of interaction with polygenic risk. The results obtained in this study contribute to our understanding of the genetic and non-genetic components of IBD risk and serve as a platform for future investigations into the disease’s complex pathogenesis.

## Supplementary Information

Supplementary Information.

## Data Availability

All data used in this paper is available on application to UK Biobank at https://www.ukbiobank.ac.uk/enable-your-research/apply-for-access.
