# Supplementary material for: Environmental variables and genome-environment interactions predicting IBD diagnosis in large UK cohort
Source: Sci Rep. 2022 Jun 28;12:10890. doi: 10.1038/s41598-022-13222-0 (PMC9240024; doi:10.1038/s41598-022-13222-0)
Supplement: Supplementary file 1 — Supplementary Information. [file 41598_2022_13222_MOESM1_ESM.docx]

**SUPPLEMENTAL MATERIALS**

Contents:

Supplemental Table 1: ICD-10/OPSC4 codes used

Supplemental Table 2: Overlap in diagnoses of CD, UC, and indeterminate colitis in UK Biobank

Supplemental Table 3: Comparing results for environmental associations with previous findings

Supplemental Table 4: Association of PRS with IBD in multivariate logistic regressions

Supplemental Table 5: Sensitivity analysis removing participants with conflicting diagnoses of CD and UC or a diagnosis of indeterminate colitis

Supplemental Figure 1: Cohort description by age and sex

Supplemental Figure 2: PRS modeling

Supplemental Figure 3: Environmental association results for 24h-recall dietary variables

Supplemental Figure 4: Gene-environment interaction results for 24h-recall dietary variables

Supplemental Figure 5: Robustness analyses for environmental associations

Supplemental Figure 6: Robustness analyses for PRSxE interactions

Supplemental Figure 7: Prospective sensitivity analyses for lifespan variables

Supplemental Figure 8: Sensitivity analysis removing participants with conflicting diagnoses of CD and UC or a diagnosis of indeterminate colitis

| Condition | ICD-10/OPSC4 codes | ICD-10/OPSC4 code descriptors |
| --- | --- | --- |
| Crohn’s disease | K50 | Crohn’s disease |
| Ulcerative colitis | K51 | Ulcerative colitis |
| Endoscopies | G45, G46, G54, G65, G80, H18, H25, H28 | Diagnostic fiberoptic endoscopic examination of the upper gastrointestinal tract, therapeutic endoscopic operations on duodenum, diagnostic endoscopic examination of duodenum, diagnostic endoscopic examination of jejunum, diagnostic endoscopic examination of ileum, diagnostic endoscopic examination of lower bowel using fiberoptic sigmoidoscope, diagnostic endoscopic examination of sigmoid colon using rigid sigmoidoscope |
| IBD-related surgeries | G04, G14, G17, G49, G50, G54.1, G58, G59, G64.1, G69, G70, G79.1, H06-12, H20, H23, H26, H29, H33, H34 | Open extirpation of lesion of oesophagus, fiberoptic endoscopic extirpation of lesion of oesophagus, endoscopic extirpation of lesion of oesophagus using rigid oesophagoscope, excision of duodenum, open extirpation of lesion of duodenum, endoscopic extirpation of lesion of duodenum, excision of jejunum, excision of jejunum, extirpation of lesion of jejunum, endoscopic extirpation of lesion of jejunum, excision of ileum, open extirpation of lesion of ileum, endoscopic extirpation of lesion of ileum, extended excision of right hemicolon, other excision of right hemicolon, excision of transverse colon, excision of left hemicolon, excision of sigmoid colon, other excision of colon, extirpation of lesion of colon, endoscopic extirpation of lesion of colon, endoscopic extirpation of lesion of lower bowel sing fiberoptic sigmoidoscope, endoscopic extirpation of lesion of sigmoid colon using rigid sigmoidoscope, subtotal excision of colon, excision of rectum, open extirpation of lesion of rectum |

**Supplemental Table 1:** ICD-10/OPSC4 codes used

|  | Self-reported CD | Self-reported UC | HES record of CD | HES record of UC | HES record of indeterminate colitis |
| --- | --- | --- | --- | --- | --- |
| Self-reported CD | 1134 | 23 | 908 | 189 | 11 |
| Self-reported UC |  | 1998 | 168 | 1443 | 17 |
| HES record of CD |  |  | 1720 | 410 | 26 |
| HES record of UC |  |  |  | 3159 | 45 |
| HES record of indeterminate colitis |  |  |  |  | 130 |

**Supplemental Table 2:** Overlapping diagnoses of CD, UC, and indeterminate colitis as recorded in the UK Biobank. Cells represent the number of individuals with both records.

|  | **Previous reports**  **Odds ratio (95% CI) unless otherwise noted** | | **Our analysis**  **Hazard ratio (95% CI)** | | |
| --- | --- | --- | --- | --- | --- |
|  | **CD** | **UC** | **CD** | **UC** | **Overall IBD** |
| **Red meat (beef and pork) consumption frequency*** | Pork: 1.4-4.0, consumed less vs. more than once per month.  Beef: no association detected.^1^ | Pork: 1.37–5.03, consumed less vs. more than once per month.  Beef: no association detected.^1^ | 0.94 - 1.14 | 1.00 - 1.14 | 0.98 - 1.11 |
| **Processed meat consumption frequency*** | Not well studied. | Not well studied. | 1.01 - 1.22 | 0.98 - 1.12 | 1.00 - 1.12 |
| **Fresh fruit consumption frequency*** | Some case-control studies suggest protective (0.1-0.9, consumed more than once a day vs. less than weekly). Others find no association.^2^ | No association detected.^2^ | 0.85 - 1.05 | 0.90 - 1.03 | 0.92 - 1.04 |
| **Alcohol consumption frequency*** | Equivocal findings. No association detected in prospective European cohort.^3^ | No association detected in prospective European cohort.^3^  Negative association found in case-control study among non-smokers only.^4^ | 0.79 - 0.97 | 0.89 - 1.02 | 0.88 - 0.99 |
| **24 hour fiber intake (g/day)*** | 0.29-0.69 (pooled from 7 studies, highest vs. lowest consumption).^5^ | Data pooled from 8 studies showed no significant association.^5^ | 0.34 - 1.40 | 0.64 - 1.41 | 0.60 - 1.28 |
| **24 hour fat intake (g/day)*** | At least two case-control studies find significant risk, but a prospective study does not.^6^ | Some case-control studies find significant risk, but not all.^2^ | 0.46 - 2.86 | 0.42 - 1.37 | 0.50 - 1.48 |
| **24 hour polyunsaturated fats intake (g/day)*** | Some case-control studies find significant risk (1.32-4.82, highest quartile vs. lowest quartile), but not all.^2,7^ | Some case-control studies find significant risk (1.04-1.66, trends across quartiles for linoleic acid consumption), but not all.^2,8^ | 0.44 - 1.75 | 0.63 - 1.50 | 0.55 - 1.24 |
| **24 hour saturated fats intake (g/day)*** | Most case-control studies find no significant association.^2^ | Case-control studies find no significant association.^2^ | 0.50 - 2.84 | 0.41 - 1.22 | 0.51 - 1.38 |
| **24 hour sugar intake (g/day)** | A prospective study in Italy found a crude risk ratio of 1.5-8.1.^31^ | Conflicting findings.^31,32^ | 0.38 – 1.47 | 0.94 – 2.19 | 0.75 – 1.68 |
| **24 hour alcohol intake (g/day)** | Equivocal findings. No association detected in prospective European cohort.^3^ | No association detected in prospective European cohort.^3^  Negative association found in case-control study among non-smokers only.^4^ | 0.81 – 2.00 | 0.33 – 0.95 | 0.59 – 1.24 |
| **24 hour iron intake (mg/day)*** | Not well studied. | Not well studied. | 1.11 - 4.23 | 0.62 - 1.56 | 0.95 - 2.14 |
| **24 hour calcium intake (mg/day)*** | Not well studied. | Not well studied. | 0.22 - 1.20 | 0.73 - 1.67 | 0.62 - 1.40 |
| **24 hour potassium intake (mg/day)*** | Not well studied. | Not well studied. | 0.27 - 1.34 | 0.55 - 1.47 | 0.46 - 1.18 |
| **24 hour magnesium intake (mg/day)*** | Not well studied. | Not well studied. | 0.22 - 1.32 | 0.45 - 1.32 | 0.42 - 1.16 |
| **24 hour protein intake (g/day)*** | Not well studied. | Not well studied. | 0.26 - 1.53 | 0.58 - 1.61 | 0.55 - 1.42 |
| **24 hour Vitamin B6 intake (mg/day)*** | Not well studied. | Not well studied. | 0.70 - 2.55 | 0.75 - 1.74 | 0.82 - 1.77 |
| **24 hour folate intake (ug/day)*** | Not well studied. | Not well studied. | 0.67 - 2.28 | 0.69 - 1.52 | 0.80 - 1.63 |
| **24 hour Vitamin B12 intake (mg/day)*** | Not well studied. | Not well studied. | 0.54 - 1.83 | 0.59 - 1.32 | 0.64 - 1.34 |
| **24 hour Vitamin C intake (mg/day)*** | Not well studied. | Not well studied. | 0.57 - 1.84 | 0.88 - 1.61 | 0.90 - 1.59 |
| **24 hour Vitamin D intake (mg/day)*** | 0.3-0.99 (hazard ratio)^9^ | Not well studied. | 0.81 - 1.95 | 0.66 - 1.41 | 0.73 - 1.43 |
| **24 hour Vitamin E intake (mg/day)*** | Not well studied. | Not well studied. | 0.40 - 1.56 | 0.57 - 1.41 | 0.63 - 1.38 |
| **Latitude at recruitment*** | 0.3-0.77 (hazard ratio) in a prospective study of women in southern vs. northern US.^10^  1.16-1.60 in northeastern vs. southern US.^11^ | 0.42-0.90 (hazard ratio) in a prospective study of women in southern vs. northern US.^10^  1.07-1.61 in northeastern vs. southern US.^11^ | 0.99 - 1.21 | 0.95 - 1.10 | 0.98 - 1.11 |
| **Latitude at birth*** | Not well studied. | Not well studied. | 0.98 - 1.14 | 0.98 - 1.10 | 1.00 - 1.09 |
| **Sun exposure during summer*** | A negative association has been suggested, but not studied.^12^ | A negative association has been suggested, but not studied.^12^ | 1.02 - 1.25 | 0.99 - 1.14 | 1.03 - 1.16 |
| **Sun exposure during winter*** | A negative association has been suggested, but not measured.^12^ | A negative association has been suggested, but not measured.^12^ | 0.93 - 1.13 | 0.97 - 1.11 | 0.98 - 1.11 |
| **Socioeconomic deprivation (IMD)*** | Very few studies of association with disease onset. | Very few studies of association with disease onset. | 1.07-1.26 | 1.12-1.26 | 1.14-1.26 |
| **Breastfed as a baby** | 0.26-0.79 in meta-analysis of case-control studies.^13^  1.1-2.1 (absence of breastfeeding vs. presence).^14^ | 0.38-0.81 in meta-analysis of mostly case-control studies.^13^  2.1 (relative risk).^15^  1.1-3.3 (absence of breastfeeding vs. presence)^14^ | 0.84 - 1.11 | 0.97 - 1.21 | 0.94 - 1.12 |
| **Birth by cesarean section** | 1.12-1.70 in meta-analysis of 6 case-control studies.^16^ But no association detected in Canadian case-control study.^17^ | No association detected.^16^ | 0.70 - 2.21 | 0.58 - 1.51 | 0.72 - 1.48 |
| **Maternal smoking around birth** | No association detected.^18^ | No association detected.^18^ | 0.97 - 1.23 | 0.99 - 1.19 | 1.02 - 1.17 |
| **Appendectomy** | Incidence rate ratio: 1.21-3.79^19^  Meta-analysis finds elevated risk 1-4 years after operation (1.66-2.38, relative risk).^20^ | 0.38-0.87.^21^  Other studies also find protective effect.^22,23^ | 0.77 – 1.14 | 0.29 – 0.45 | 0.51 – 0.67 |
| **Long-term childhood antibiotics** | Case-control studies suggest increased risk 2-5 years after antibiotics exposure (1.18–1.40), but effect of childhood antibiotics on later-onset CD is not well studied.^24^ | Case-control studies suggest increased risk 2-5 years after antibiotics exposure (1.16–1.36), but effect of childhood antibiotics on later-onset UC is not well studied.^24^ | 1.13 - 2.01 | 0.94 - 1.47 | 1.11 - 1.56 |
| **NSAIDs** | Relationship between pre-disease exposure and CD onset not well studied.  Cohort study in women found that high NSAIDs use is weakly associated with disease (0.99-2.56, hazard ratio).^25^ | Relationship between pre-disease exposure and CD onset not well studied.  Cohort study in women found that high NSAIDs use is linked to UC (1.16-2.99, hazard ratio).^25^ | 1.00 - 1.49 | 0.91 - 1.20 | 0.99 - 1.26 |
| **Oral contraceptive therapy (current use)** | No association detected in 2 UK cohort studies.^26,27^ A US cohort study in women finds current use is a risk (1.65-4.52) as well as former use (1.05-1.85).^28^ | No association detected in 2 UK cohort studies.^26,27^ A US cohort study in women finds association only for those with smoking history (1.13-2.35).^28^ | 0.92 – 1.53 | 0.79 – 1.22 | 0.89 – 1.24 |
| **Oral contraceptive therapy (previous use)** | No association detected in 2 UK cohort studies.^26,27^ A US cohort study in women finds current use is a risk (1.65-4.52) as well as former use (1.05-1.85).^28^ | No association detected in 2 UK cohort studies.^26,27^ A US cohort study in women finds association only for those with smoking history (1.13-2.35).^28^ | 0.84 – 1.34 | 0.81 – 1.18 | 0.88 – 1.18 |
| **Smoking (current use)** | Current users are at risk (1.42-2.53), as are former users (1.05-1.73).^29^ | Current users are protected (0.61-1.20), but former users are at risk (1.26-1.93).^29^ | 1.49 – 2.00 | 0.52 – 0.71 | 0.88 – 1.08 |
| **Smoking (previous use)** | Current users are at risk (1.42-2.53), as are former users (1.05-1.73).^29^ | Current users are protected (0.61-1.20), but former users are at risk (1.26-1.93).^29^ | 1.45 – 2.10 | 2.36 – 2.98 | 2.08 – 2.52 |
| **Hormone replacement therapy (current use)** | No association detected.^30^ | Prospective cohort study found elevated risk (HR: 1.07-2.74).^30^ | Not well-modeled | Not well-modeled | Not well-modeled |
| **Hormone replacement therapy (previous use)** | Not well-studied. | Not well-studied. | Not well-modeled | Not well-modeled | Not well-modeled |

* = HR given per standard deviation of the variable

**Supplemental Table 3**: Comparison of the association between IBD onset and environmental exposures in the UK Biobank and in previously published findings

| **Disease** | **N** | **Odds Ratio (per standard deviation PRS)** | **95% CI** | **p-value** |
| --- | --- | --- | --- | --- |
| IBD | 364898 | 1.67 | 1.29-2.16 | p<0.001 |
| CD | 364898 | 1.72 | 1.65-1.80 | p<0.001 |
| UC | 364898 | 1.82 | 1.76-1.88 | p<0.001 |

**Supplemental Table 4**: Association of PRS with IBD diagnosis in UK Biobank using multivariate logistic regressions

|  | **Main results**  Hazard ratio (95% CI) | | | **After removing those with conflicting histories of CD and UC or diagnosis of indeterminate colitis**  Hazard ratio (95% CI) | | |
| --- | --- | --- | --- | --- | --- | --- |
|  | **CD** | **UC** | **IBD** | **CD** | **UC** | **IBD** |
| **Red meat (beef and pork) consumption frequency*** | 0.94 - 1.14 | 1.00 - 1.14 | 0.98 - 1.11 | 0.93 - 1.16 | 0.98 - 1.13 | 0.98 - 1.11 |
| **Processed meat consumption frequency*** | 1.01 - 1.22 | 0.98 - 1.12 | 1.00 - 1.12 | 1 - 1.23 | 0.96 - 1.11 | 0.99 - 1.12 |
| **Fresh fruit consumption frequency*** | 0.85 - 1.05 | 0.90 - 1.03 | 0.92 - 1.04 | 0.82 - 1.05 | 0.91 - 1.06 | 0.91 - 1.04 |
| **Alcohol consumption frequency*** | 0.79 - 0.97 | 0.89 - 1.02 | 0.88 - 0.99 | 0.78 - 0.98 | 0.9 - 1.03 | 0.88 - 0.99 |
| **24 hour fiber intake (g/day)*** | 0.34 - 1.40 | 0.64 - 1.41 | 0.60 - 1.28 | 0.3 - 1.34 | 0.6 - 1.51 | 0.57 - 1.27 |
| **24 hour fat intake (g/day)*** | 0.46 - 2.86 | 0.42 - 1.37 | 0.50 - 1.48 | 0.35 - 2.48 | 0.45 - 1.73 | 0.5 - 1.54 |
| **24 hour polyunsaturated fats intake (g/day)*** | 0.44 - 1.75 | 0.63 - 1.50 | 0.55 - 1.24 | 0.29 - 1.41 | 0.55 - 1.5 | 0.49 - 1.15 |
| **24 hour saturated fats intake (g/day)*** | 0.50 - 2.84 | 0.41 - 1.22 | 0.51 - 1.38 | 0.46 - 2.88 | 0.45 - 1.54 | 0.56 - 1.55 |
| **24 hour sugar intake (g/day)** | 0.38 – 1.47 | 0.94 – 2.19 | 0.75 – 1.68 | 0.27 - 1.35 | 0.97 - 2.51 | 0.81 - 1.83 |
| **24 hour alcohol intake (g/day)** | 0.81 – 2.00 | 0.33 – 0.95 | 0.59 – 1.24 | 0.82 - 2.14 | 0.31 - 1.03 | 0.58 - 1.24 |
| **24 hour iron intake (mg/day)*** | 1.11 - 4.23 | 0.62 - 1.56 | 0.95 - 2.14 | 1.02 - 4.27 | 0.65 - 1.87 | 0.93 - 2.18 |
| **24 hour calcium intake (mg/day)*** | 0.22 - 1.20 | 0.73 - 1.67 | 0.62 - 1.40 | 0.25 - 1.45 | 0.78 - 1.9 | 0.68 - 1.5 |
| **24 hour potassium intake (mg/day)*** | 0.27 - 1.34 | 0.55 - 1.47 | 0.46 - 1.18 | 0.19 - 1.21 | 0.44 - 1.43 | 0.43 - 1.15 |
| **24 hour magnesium intake (mg/day)*** | 0.22 - 1.32 | 0.45 - 1.32 | 0.42 - 1.16 | 0.21 - 1.41 | 0.42 - 1.44 | 0.43 - 1.23 |
| **24 hour protein intake (g/day)*** | 0.26 - 1.53 | 0.58 - 1.61 | 0.55 - 1.42 | 0.24 - 1.59 | 0.6 - 1.89 | 0.57 - 1.54 |
| **24 hour Vitamin B6 intake (mg/day)*** | 0.70 - 2.55 | 0.75 - 1.74 | 0.82 - 1.77 | 0.56 - 2.31 | 0.74 - 1.97 | 0.76 - 1.62 |
| **24 hour folate intake (ug/day)*** | 0.67 - 2.28 | 0.69 - 1.52 | 0.80 - 1.63 | 0.64 - 2.34 | 0.62 - 1.57 | 0.82 - 1.83 |
| **24 hour Vitamin B12 intake (mg/day)*** | 0.54 - 1.83 | 0.59 - 1.32 | 0.64 - 1.34 | 0.32 - 1.69 | 0.57 - 1.45 | 0.61 - 1.34 |
| **24 hour Vitamin C intake (mg/day)*** | 0.57 - 1.84 | 0.88 - 1.61 | 0.90 - 1.59 | 0.53 - 1.85 | 0.89 - 1.74 | 0.89 - 1.62 |
| **24 hour Vitamin D intake (mg/day)*** | 0.81 - 1.95 | 0.66 - 1.41 | 0.73 - 1.43 | 0.41 - 1.86 | 0.51 - 1.42 | 0.57 - 1.32 |
| **24 hour Vitamin E intake (mg/day)*** | 0.40 - 1.56 | 0.57 - 1.41 | 0.63 - 1.38 | 0.29 - 1.48 | 0.56 - 1.53 | 0.57 - 1.32 |
| **Latitude at recruitment*** | 0.99 - 1.21 | 0.95 - 1.10 | 0.98 - 1.11 | 0.99 - 1.25 | 0.93 - 1.08 | 0.97 - 1.11 |
| **Latitude at birth*** | 0.98 - 1.14 | 0.98 - 1.10 | 1.00 - 1.09 | 0.97 - 1.14 | 0.98 - 1.1 | 1 - 1.1 |
| **Sun exposure during summer*** | 1.02 - 1.25 | 0.99 - 1.14 | 1.03 - 1.16 | 0.92 - 1.14 | 0.98 - 1.13 | 0.98 - 1.11 |
| **Sun exposure during winter*** | 0.93 - 1.13 | 0.97 - 1.11 | 0.98 - 1.11 | 1.03 - 1.3 | 1 - 1.16 | 1.04 - 1.18 |
| **Socioeconomic deprivation (IMD)*** | 1.07-1.26 | 1.12-1.26 | 1.14-1.26 | 1.07 - 1.29 | 1.11 - 1.26 | 1.13 - 1.25 |
| **Breastfed as a baby** | 0.84 - 1.11 | 0.97 - 1.21 | 0.94 - 1.12 | 0.93 - 1.03 | 0.98 - 1.05 | 0.97 - 1.03 |
| **Birth by cesarean section** | 0.70 - 2.21 | 0.58 - 1.51 | 0.72 - 1.48 | 0.93 - 1.11 | 0.94 - 1.06 | 0.95 - 1.05 |
| **Maternal smoking around birth** | 0.97 - 1.23 | 0.99 - 1.19 | 1.02 - 1.17 | 0.99 - 1.1 | 1.01 - 1.08 | 1.02 - 1.08 |
| **Appendectomy** | 0.77 – 1.14 | 0.29 – 0.45 | 0.51 – 0.67 | 0.74 - 1.15 | 0.29 - 0.46 | 0.5 - 0.67 |
| **Long-term childhood antibiotics** | 1.13 - 2.01 | 0.94 - 1.47 | 1.11 - 1.56 | 1 - 1.26 | 0.98 - 1.15 | 1.02 - 1.16 |
| **NSAIDs** | 1.00 - 1.49 | 0.91 - 1.20 | 0.99 - 1.26 | 1 - 1.21 | 0.96 - 1.1 | 0.99 - 1.11 |
| **Oral contraceptive therapy (current use)** | 0.92 – 1.53 | 0.79 – 1.22 | 0.89 – 1.24 | 0.87 - 1.52 | 0.8 - 1.19 | 0.89 - 1.21 |
| **Oral contraceptive therapy (previous use)** | 0.84 – 1.34 | 0.81 – 1.18 | 0.88 – 1.18 | 0.89 - 1.57 | 0.81 - 1.26 | 0.89 - 1.26 |
| **Smoking (current use)** | 1.49 – 2.00 | 0.52 – 0.71 | 0.88 – 1.08 | 1.42 - 2.16 | 2.35 - 3 | 2.08 - 2.56 |
| **Smoking (previous use)** | 1.45 – 2.10 | 2.36 – 2.98 | 2.08 – 2.52 | 1.54 - 2.13 | 0.51 - 0.71 | 0.88 - 1.09 |
| **Hormone replacement therapy (current use)** | Not well-modeled | Not well-modeled | Not well-modeled | Not well-modeled | Not well-modeled | Not well-modeled |
| **Hormone replacement therapy (previous use)** | Not well-modeled | Not well-modeled | Not well-modeled | Not well-modeled | Not well-modeled | Not well-modeled |

**Supplemental Table 5**: Comparison of main results (95% CI for hazard ratios) to the results obtained after removing participants with conflicting diagnoses of CD and UC or a diagnosis of indeterminate colitis. In both the main analysis and sensitivity analysis, the same Cox regressions were used.


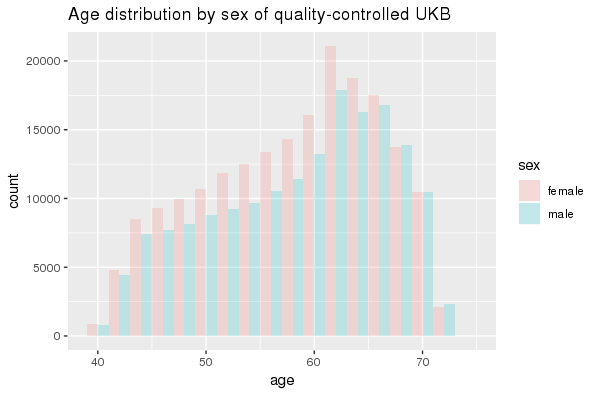

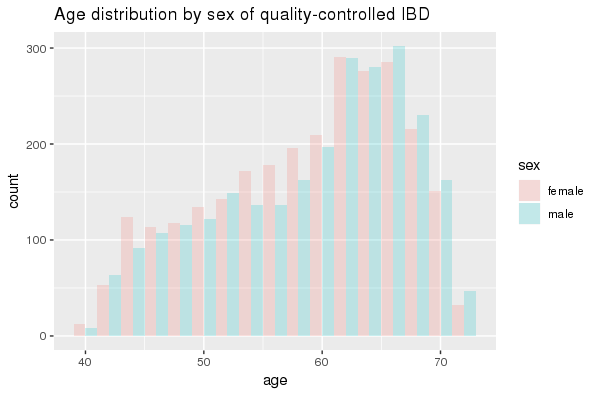

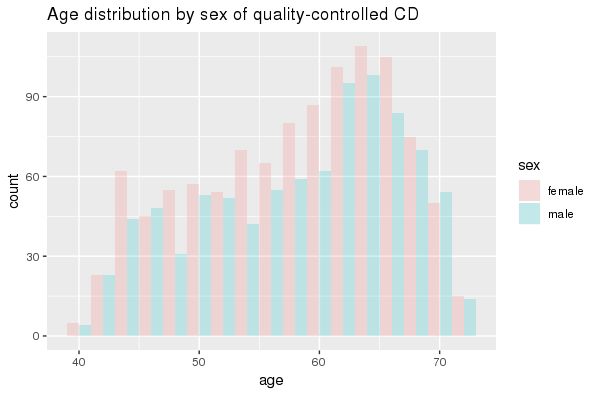


(a)

(b)

(d)

(c)


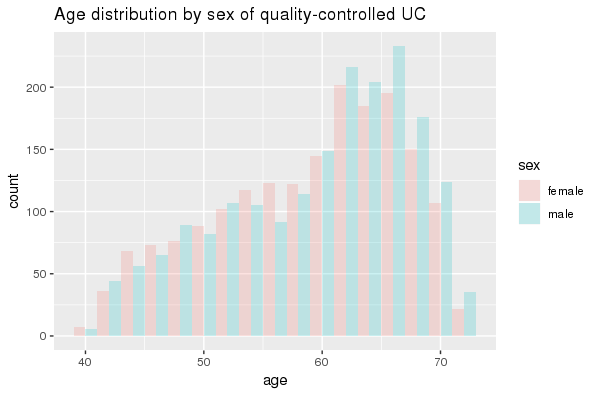


**Supplemental Figure 1:** Age distribution stratified by sex for the quality-controlled cohort and the three disease subpopulations.


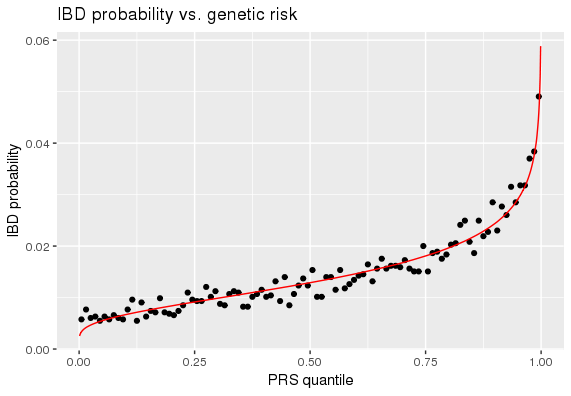


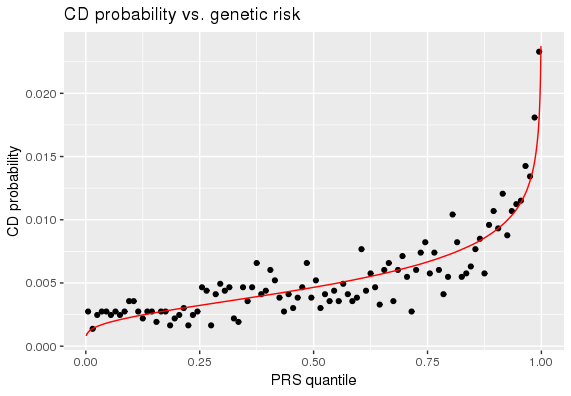


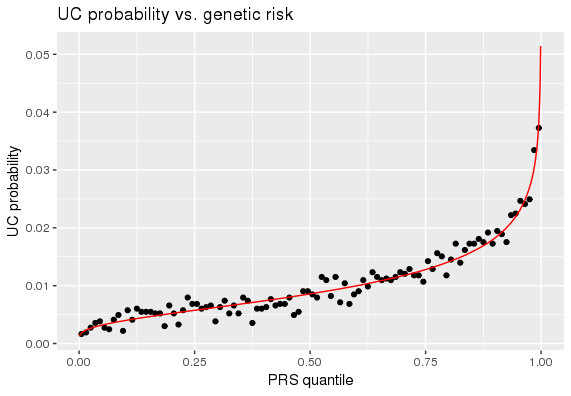


**Supplemental Figure 2:** IBD probability plotted against PRS quartiles (black dots) for each disease along with the logistic models (red line).


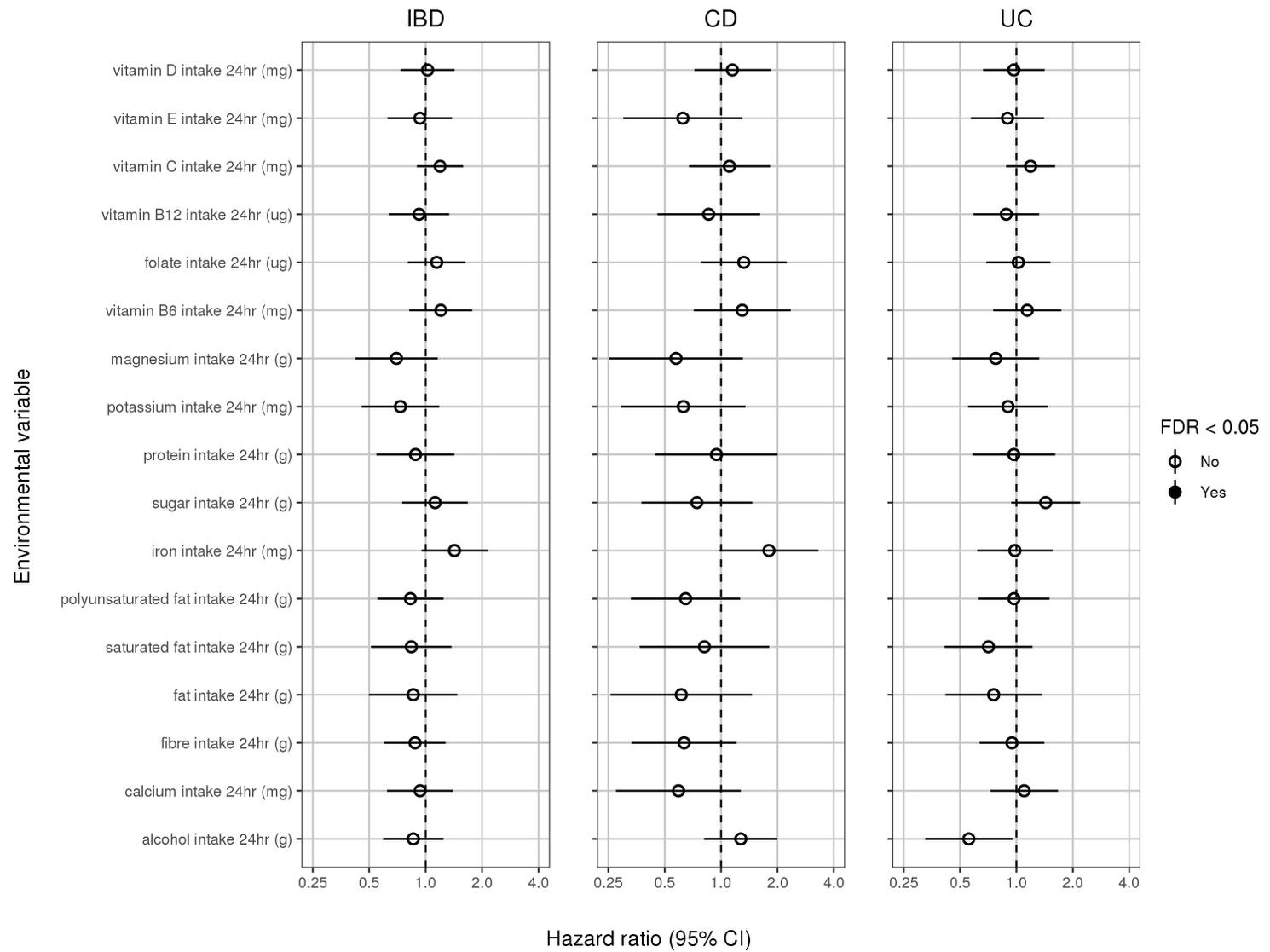


**Supplemental Figure 3:** Forest plot of hazard ratios (dots) and 95% confidence intervals (lines) obtained from Cox regressions for the 24h-recall dietary variables. Hazard ratios were adjusted for other covariates, including polygenic risk. Statistically significant results (FDR < 0.05) represented by filled circles. Hazard ratios are given per standard deviation of the variable.


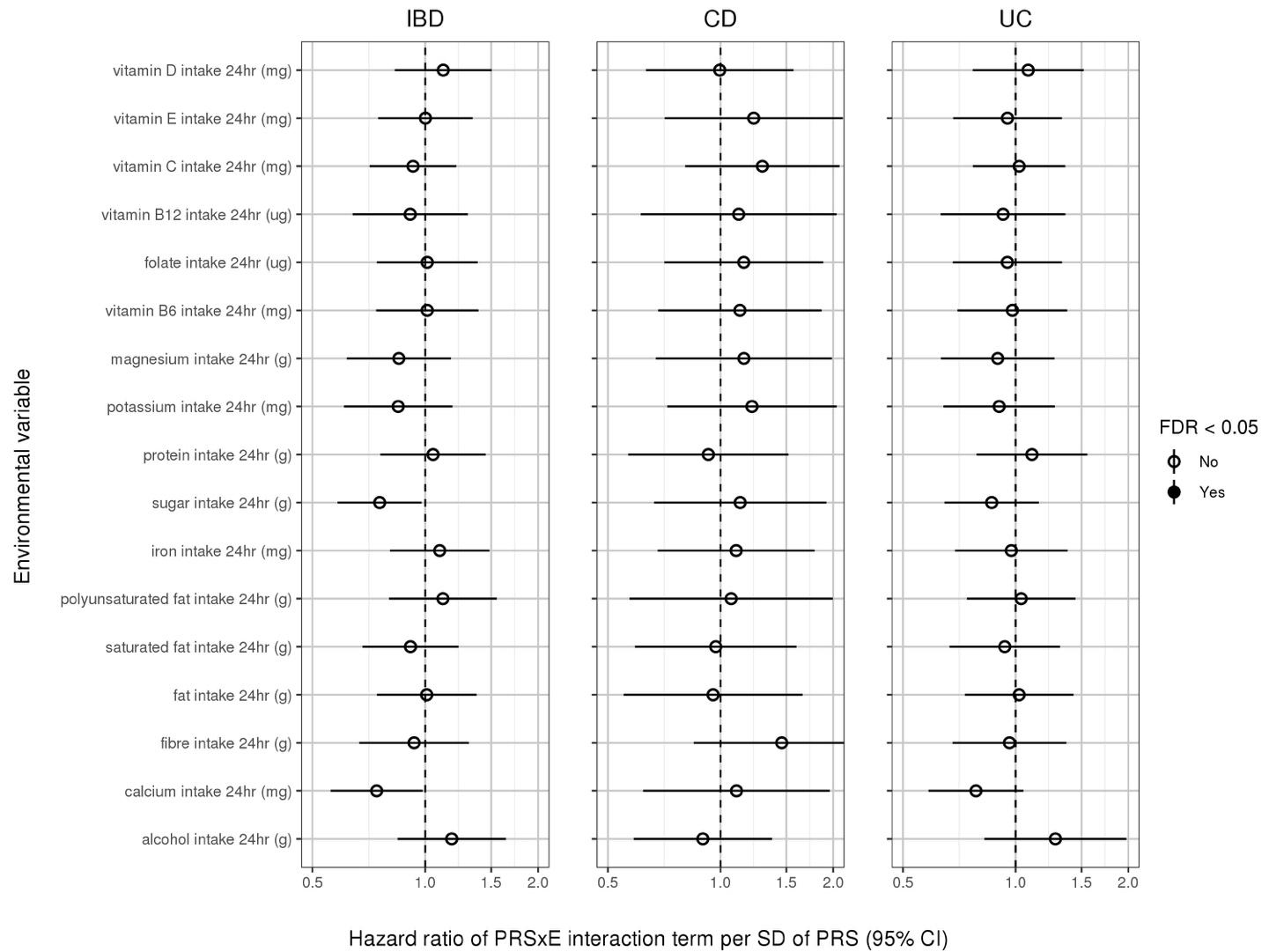


**Supplemental Figure 4:** Forest plot of hazard ratios (dots) and 95% confidence intervals (lines) for PRSxE interactions obtained from Cox regressions for the 24h-recall dietary variables. Hazard ratios were adjusted for other covariates, including polygenic risk. Statistically significant results (FDR < 0.05) represented by filled circles. x-axis truncated at 2. Hazard ratios are given per standard deviation of the variable per standard deviation of PRS.


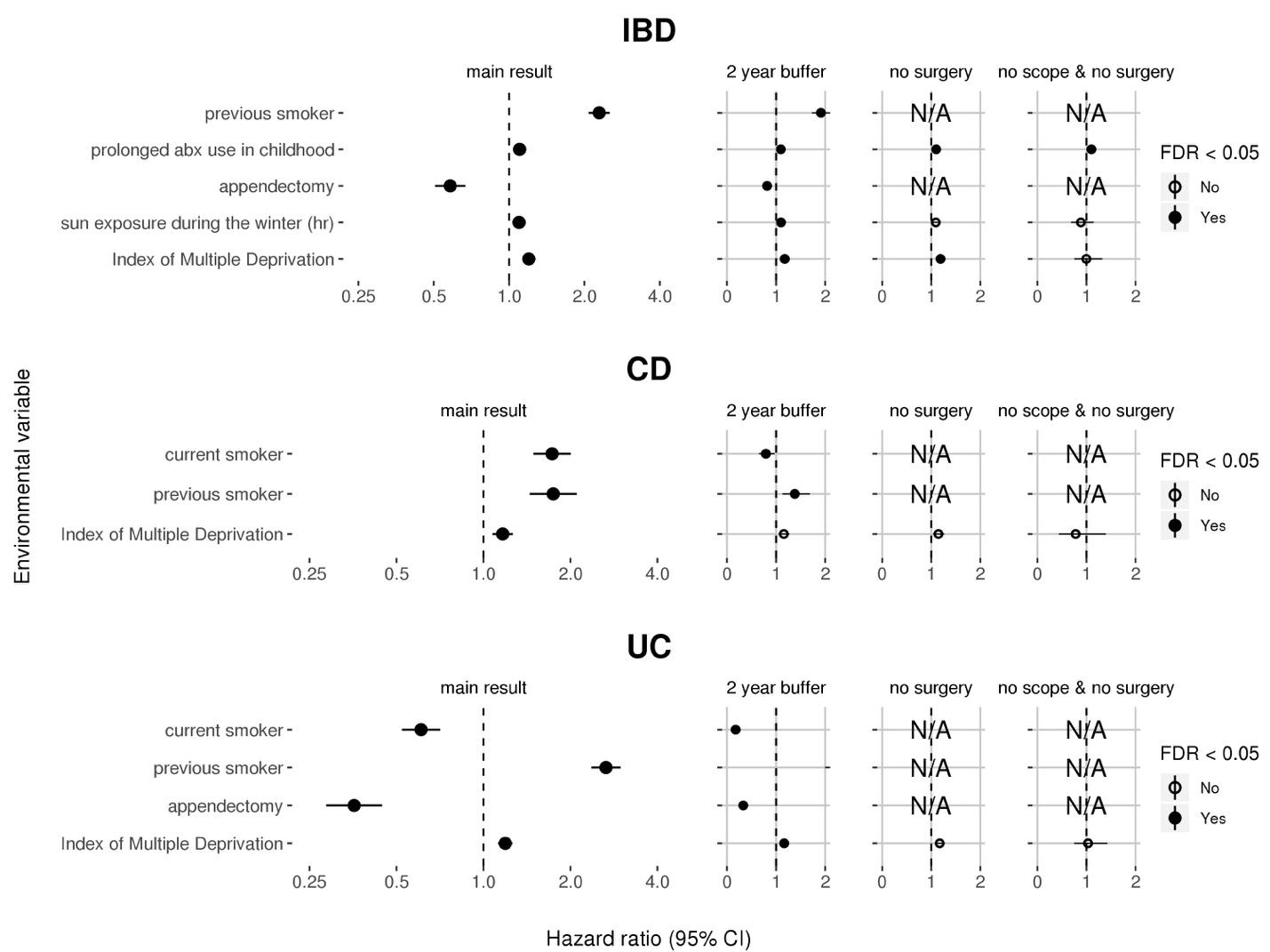


**Supplemental Figure 5:** Forest plot of hazard ratios (95% confidence intervals) obtained from Cox regressions in different robustness analyses. Given as a 95% confidence interval of the hazard ratio. Statistically significant results (FDR < 0.05) are shown as filled circles. x-axis truncated at 3.0 in left-most panels. In the “2 year buffer” analysis, participants getting IBD within two years of truncation point or within two years of a change in environmental status were removed from analysis. In the “no surgery” robustness analysis, participants who had undergone IBD-related surgeries before the truncation point were removed from analysis. Similarly, in the “no scope & no surgery” analysis, participants who had undergone IBD-related surgeries or endoscopies before truncation were removed from analysis. Analyses which did not feature truncation were only tested with the “2 year buffer” analysis. For continuous variables, hazard ratios were scaled by the standard deviation of the variable. “N/A” indicates robustness analysis not performed based on whether the primary analysis was prospective or retrospective (see Methods).


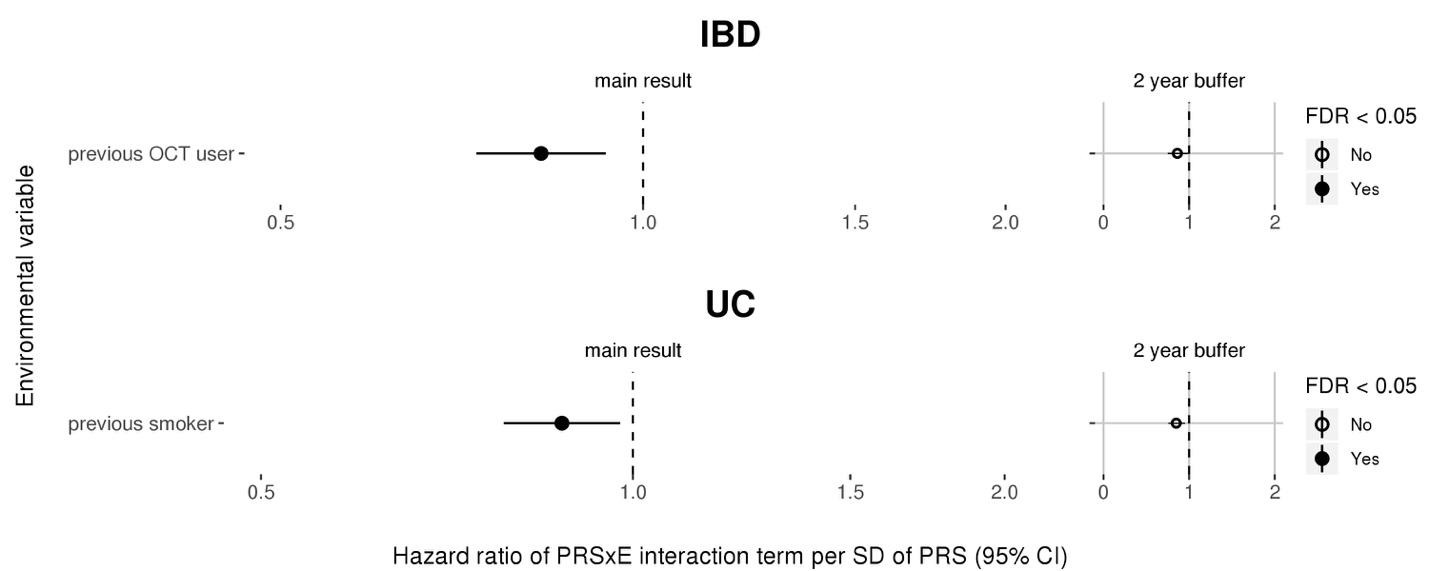


**Supplemental Figure 6:** Forest plot of hazard ratios (95% confidence intervals) for PRSxE interaction obtained from Cox regressions in different robustness analyses. Given as a 95% confidence interval of the hazard ratio. Statistically significant results (FDR < 0.05) are shown as filled circles. In the “2 year buffer” analysis, participants getting IBD within two years of a change in environmental status were removed from analysis.


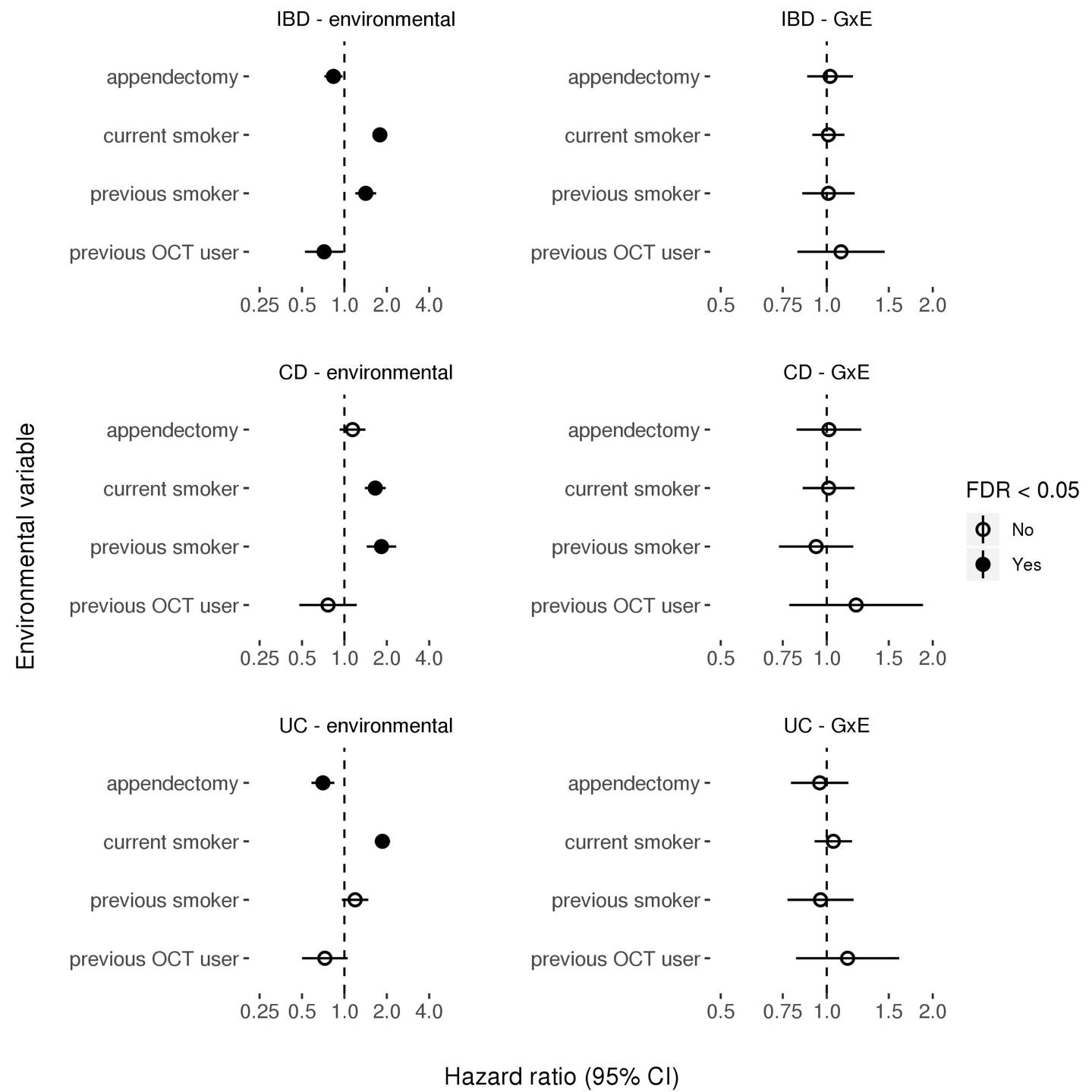


**Supplemental Figure 7:** Results of the prospective validations of the primary retrospective analyses for lifespan variables. Forest plot of hazard ratios (dots) and 95% confidence intervals (lines) obtained from Cox regressions. Hazard ratios were adjusted for other covariates, including polygenic risk (see Methods). Statistically significant results (FDR < 0.05) represented by filled circles. In these prospective sensitivity analyses, follow-up began at recruitment and IBD status was determined through HES records only.

(a) IBD


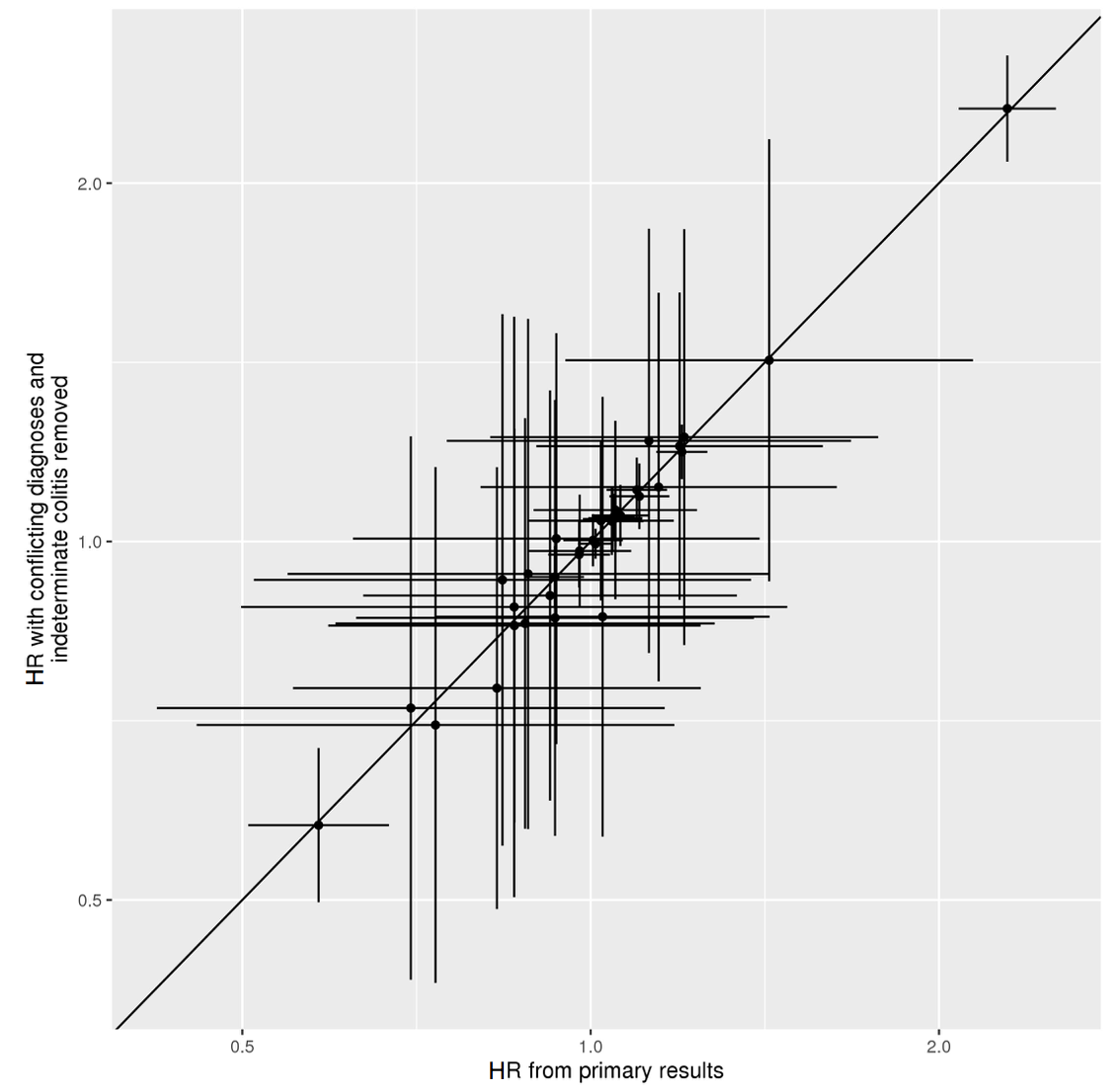


(b) CD


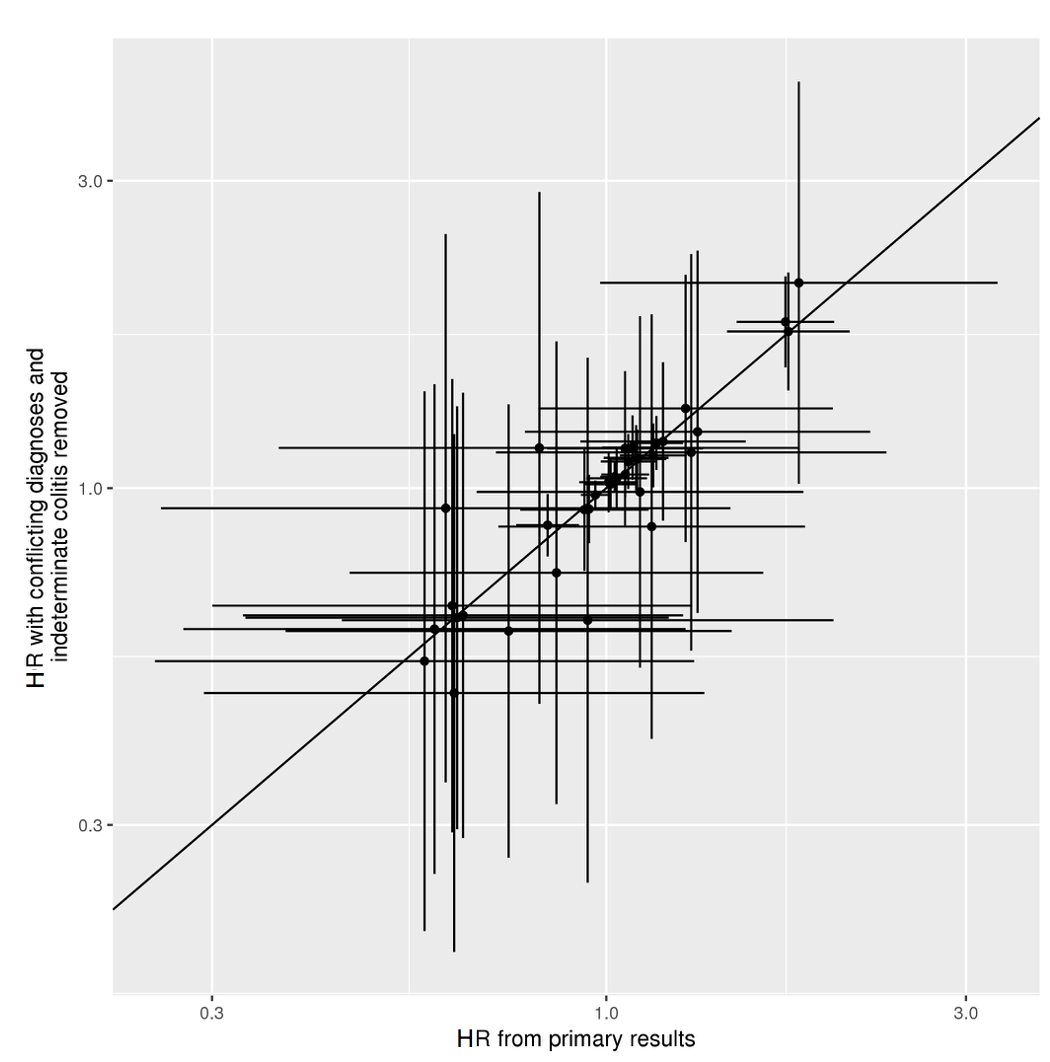


(c) UC


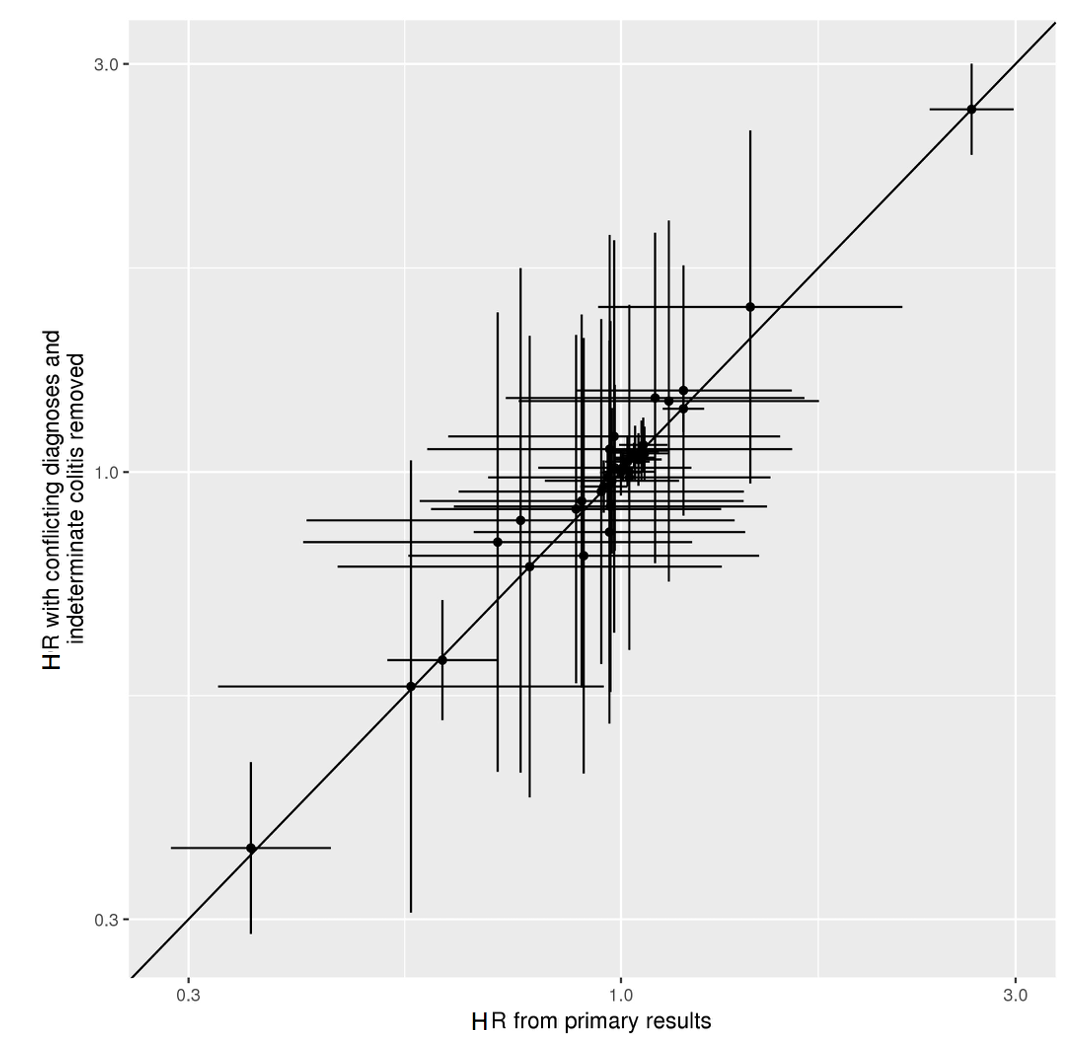


**Supplemental Figure 8**: Comparison of main results (95% CI for hazard ratios, horizontal lines) to the results obtained after excluding participants with conflicting diagnoses of CD and UC or a diagnosis of indeterminate colitis (vertical lines). In both the main analysis and the sensitivity analysis, the same Cox regressions were used to evaluate the association between environmental factors and IBD diagnosis (results for IBD in panel a, CD in panel b, UC in panel c). A y=x line is drawn for reference.

**SUPPLEMENTAL REFERENCES**

1. Bernstein CN, Rawsthorne P, Cheang M, Blanchard JF. A population-based case control study of potential risk factors for IBD. Am J Gastroenterol. United States; 2006 May;101(5):993–1002. PMID: 16696783

2. Hou JK, Abraham B, El-Serag H. Dietary intake and risk of developing inflammatory bowel disease: a systematic review of the literature. Am J Gastroenterol. United States; 2011 Apr;106(4):563–573. PMID: 21468064

3. Bergmann MM, Hernandez V, Bernigau W, Boeing H, Chan SSM, Luben R, Khaw K-T, van Schaik F, Oldenburg B, Bueno-de-Mesquita B, Overvad K, Palli D, Masala G, Carbonnel F, Boutron-Ruault M-C, Olsen A, Tjonneland A, Kaaks R, Katzke V, Riboli E, Hart AR. No association of alcohol use and the risk of ulcerative colitis or Crohn’s disease: data from a European Prospective cohort study (EPIC). Eur J Clin Nutr [Internet]. 2017 Apr 25;71(4):512–518. Available from: http://www.nature.com/articles/ejcn2016271

4. Boyko EJ, Perera DR, Koepsell TD, Keane EM, Inui TS. Coffee and alcohol use and the risk of ulcerative colitis. Am J Gastroenterol. United States; 1989 May;84(5):530–534. PMID: 2719009

5. Liu X, Wu Y, Li F, Zhang D. Dietary fiber intake reduces risk of inflammatory bowel disease: result from a meta-analysis. Nutr Res. United States; 2015 Sep;35(9):753–758. PMID: 26126709

6. Jantchou P, Morois S, Clavel-Chapelon F, Boutron-Ruault M-C, Carbonnel F. Animal protein intake and risk of inflammatory bowel disease: The E3N prospective study. Am J Gastroenterol. United States; 2010 Oct;105(10):2195–2201. PMID: 20461067

7. Amre DK, D’Souza S, Morgan K, Seidman G, Lambrette P, Grimard G, Israel D, Mack D, Ghadirian P, Deslandres C, Chotard V, Budai B, Law L, Levy E, Seidman EG. Imbalances in dietary consumption of fatty acids, vegetables, and fruits are associated with risk for Crohn’s disease in children. Am J Gastroenterol. United States; 2007 Sep;102(9):2016–2025. PMID: 17617201

8. Investigators I in ES, Tjonneland A, Overvad K, Bergmann MM, Nagel G, Linseisen J, Hallmans G, Palmqvist R, Sjodin H, Hagglund G, Berglund G, Lindgren S, Grip O, Palli D, Day NE, Khaw K-T, Bingham S, Riboli E, Kennedy H, A Hart. Linoleic acid, a dietary n-6 polyunsaturated fatty acid, and the aetiology of ulcerative colitis: a nested case-control study within a European prospective cohort study. Gut [Internet]. 2009 Dec 1;58(12):1606–1611. Available from: http://gut.bmj.com/cgi/doi/10.1136/gut.2008.169078

9. Ananthakrishnan AN, Khalili H, Higuchi LM, Bao Y, Korzenik JR, Giovannucci EL, Richter JM, Fuchs CS, Chan AT. Higher predicted vitamin D status is associated with reduced risk of Crohn’s disease. Gastroenterology [Internet]. 2012 Mar;142(3):482–9. Available from: http://www.ncbi.nlm.nih.gov/pubmed/22155183 PMID: 22155183

10. Khalili H, Huang ES, Ananthakrishnan AN, Higuchi L, Richter JM, Fuchs CS, Chan AT. Geographical variation and incidence of inflammatory bowel disease among US women. Gut [Internet]. 2012 Dec;61(12):1686–92. Available from: http://www.ncbi.nlm.nih.gov/pubmed/22241842 PMID: 22241842

11. Kappelman MD, Rifas-Shiman SL, Kleinman K, Ollendorf D, Bousvaros A, Grand RJ, Finkelstein JA. The prevalence and geographic distribution of Crohn’s disease and ulcerative colitis in the United States. Clin Gastroenterol Hepatol [Internet]. 2007 Dec;5(12):1424–9. Available from: http://www.ncbi.nlm.nih.gov/pubmed/17904915 PMID: 17904915

12. Palmer MT, Weaver CT. Linking vitamin d deficiency to inflammatory bowel disease. Inflamm Bowel Dis. England; 2013 Sep;19(10):2245–2256. PMID: 23591600

13. Klement E, Cohen R V, Boxman J, Joseph A, Reif S. Breastfeeding and risk of inflammatory bowel disease: a systematic review with meta-analysis. Am J Clin Nutr. United States; 2004 Nov;80(5):1342–1352. PMID: 15531685

14. Corrao G, Tragnone A, Caprilli R, Trallori G, Papi C, Andreoli A, Di Paolo M, Riegler G, Rigo GP, Ferrau O, Mansi C, Ingrosso M, Valpiani D. Risk of inflammatory bowel disease attributable to smoking, oral contraception and breastfeeding in Italy: a nationwide case-control study. Cooperative Investigators of the Italian Group for the Study of the Colon and the Rectum (GISC). Int J Epidemiol. England; 1998 Jun;27(3):397–404. PMID: 9698126

15. Acheson ED, True Love SC. Early weaning in the aetiology of ulcerative colitis. A study of feeding in infancy in cases and controls. Br Med J. England; 1961 Oct;2(5257):929–933. PMID: 13681206

16. Li Y, Tian Y, Zhu W, Gong J, Gu L, Zhang W, Guo Z, Li N, Li J. Cesarean delivery and risk of inflammatory bowel disease: a systematic review and meta-analysis. Scand J Gastroenterol. England; 2014 Jul;49(7):834–844. PMID: 24940636

17. Bernstein CN, Banerjee A, Targownik LE, Singh H, Ghia JE, Burchill C, Chateau D, Roos LL. Cesarean Section Delivery Is Not a Risk Factor for Development of Inflammatory Bowel Disease: A Population-based Analysis. Clin Gastroenterol Hepatol. United States; 2016 Jan;14(1):50–57. PMID: 26264640

18. Rigas A, Rigas B, Glassman M, Yen YY, Lan SJ, Petridou E, Hsieh CC, Trichopoulos D. Breast-feeding and maternal smoking in the etiology of Crohn’s disease and ulcerative colitis in childhood. Ann Epidemiol. United States; 1993 Jul;3(4):387–392. PMID: 8275215

19. Andersson RE, Olaison G, Tysk C, Ekbom A. Appendectomy is followed by increased risk of Crohn’s disease. Gastroenterology. United States; 2003 Jan;124(1):40–46. PMID: 12512028

20. Kaplan GG, Jackson T, Sands BE, Frisch M, Andersson RE, Korzenik J. The risk of developing Crohn’s disease after an appendectomy: a meta-analysis. Am J Gastroenterol. United States; 2008 Nov;103(11):2925–2931. PMID: 18775018

21. Andersson RE, Olaison G, Tysk C, Ekbom A. Appendectomy and protection against ulcerative colitis. N Engl J Med. United States; 2001 Mar;344(11):808–814. PMID: 11248156

22. Koutroubakis IE, Vlachonikolis IG, Kapsoritakis A, Spanoudakis S, Roussomoustakaki M, Mouzas IA, Kouroumalis EA, Manousos ON. Appendectomy, tonsillectomy, and risk of inflammatory bowel disease: case-controlled study in Crete. Dis Colon Rectum. United States; 1999 Feb;42(2):225–230. PMID: 10211500

23. Reif S, Lavy A, Keter D, Broide E, Niv Y, Halak A, Ron Y, Eliakim R, Odes S, Patz J, Fich A, Villa Y, Arber N, Gilat T. Appendectomy is more frequent but not a risk factor in Crohn’s disease while being protective in ulcerative colitis: a comparison of surgical procedures in inflammatory bowel disease. Am J Gastroenterol. United States; 2001 Mar;96(3):829–832. PMID: 11280559

24. Shaw SY, Blanchard JF, Bernstein CN. Association between the use of antibiotics and new diagnoses of Crohn’s disease and ulcerative colitis. Am J Gastroenterol. United States; 2011 Dec;106(12):2133–2142. PMID: 21912437

25. Ananthakrishnan AN, Higuchi LM, Huang ES, Khalili H, Richter JM, Fuchs CS, Chan AT. Aspirin, nonsteroidal anti-inflammatory drug use, and risk for Crohn disease and ulcerative colitis: a cohort study. Ann Intern Med. United States; 2012 Mar;156(5):350–359. PMID: 22393130

26. Logan R, Kay C. Oral contraception, smoking and inflammatory bowel disease—findings in the Royal College of General Practitioners Oral contraception study. Int J Epidemiol. 1989;18:105–7.

27. Vessey M, Jewell D, Smith A, Yeates D, McPherson K. Chronic inflammatory bowel disease, cigarette smoking, and use of oral contraceptives: findings in a large cohort study of women of childbearing age. Br Med J (Clin Res Ed) [Internet]. 1986 Apr 26;292(6528):1101–3. Available from: http://www.ncbi.nlm.nih.gov/pubmed/3084016 PMID: 3084016

28. Khalili H, Higuchi LM, Ananthakrishnan AN, Richter JM, Feskanich D, Fuchs CS, Chan AT. Oral contraceptives, reproductive factors and risk of inflammatory bowel disease. Gut. England; 2013 Aug;62(8):1153–1159. PMID: 22619368

29. Higuchi LM, Khalili H, Chan AT, Richter JM, Bousvaros A, Fuchs CS. A prospective study of cigarette smoking and the risk of inflammatory bowel disease in women. Am J Gastroenterol. United States; 2012 Sep;107(9):1399–1406. PMID: 22777340

30. Khalili H, Higuchi LM, Ananthakrishnan AN, Manson JE, Feskanich D, Richter JM, Fuchs CS, Chan AT. Hormone therapy increases risk of ulcerative colitis but not Crohn’s disease. Gastroenterology. United States; 2012 Nov;143(5):1199–1206. PMID: 22841783

31. Tragnone A, Valpiani D, Miglio F, Elmi G, Bazzocchi G, Pipitone E, Lanfranchi GA. Dietary habits as risk factors for inflammatory bowel disease. Eur J Gastroenterol Hepatol [Internet]. 1995 Jan;7(1):47–51. Available from: http://www.ncbi.nlm.nih.gov/pubmed/7866810 PMID: 7866810

32. Ananthakrishnan AN. Impact of Diet on Risk of IBD. Crohn’s Colitis 360 [Internet]. 2020 Jan 1;2(1). Available from: https://academic.oup.com/crohnscolitis360/article/doi/10.1093/crocol/otz054/5707521
